# Mechanisms and Applications of Manganese-Based Nanomaterials in Tumor Diagnosis and Therapy

**DOI:** 10.34133/bmr.0158

**Published:** 2025-02-28

**Authors:** Xiaowen Ma, Chuan He, Yang Wang, Xingrui Cao, Zikai Jin, Yi Ge, Zhipeng Cao, Mingxin An, Liang Hao

**Affiliations:** ^1^ Department of Chemistry, School of Forensic Medicine, China Medical University, Shenyang 110122, China.; ^2^ Liaoning Province Key Laboratory of Forensic Bio-evidence Sciences, Shenyang 110122, China.; ^3^ China Medical University Center of Forensic Investigation, Shenyang 110122, China.; ^4^ First Department of Clinical Medicine, China Medical University, Shenyang 110122, China.; ^5^ Department of Laboratory Medicine, the First Hospital of China Medical University, Shenyang 110001, China.; ^6^ School of Pharmacy, Queen’s University Belfast, Belfast BT9 7BL, UK.; ^7^ Department of Forensic Pathology, School of Forensic Medicine, China Medical University, Shenyang 110122, China.; ^8^ Department of Developmental Cell Biology, Key Laboratory of Cell Biology, Ministry of Public Health, and Key Laboratory of Medical Cell Biology, Ministry of Education, China Medical University, Shenyang 110122, China.

## Abstract

Tumors are the second most common cause of mortality globally, ranking just below heart disease. With continuous advances in diagnostic technology and treatment approaches, the survival rates of some cancers have increased. Nevertheless, due to the complexity of the mechanisms underlying tumors, cancer remains a serious public health issue that threatens the health of the population globally. Manganese (Mn) is an essential trace element for the human body. Its regulatory role in tumor biology has received much attention in recent years. Developments in nanotechnology have led to the emergence of Mn-based nanoparticles that have great potential for use in the diagnosis and treatment of cancers. Mn-based nanomaterials can be integrated with conventional techniques, including chemotherapy, radiation therapy, and gene therapy, to augment their therapeutic effectiveness. Further, Mn-based nanomaterials can play a synergistic role in emerging treatment strategies for tumors, such as immunotherapy, photothermal and photodynamic therapy, electromagnetic hyperthermia, sonodynamic therapy, chemodynamic therapy, and intervention therapy. Moreover, Mn-based nanomaterials can enhance both the precision of tumor diagnostics and the capability for combined diagnosis and treatment. This article examines the roles and associated mechanisms of Mn in the field of physiology and tumor biology, with a focus on the application prospects of Mn-based nanomaterials in tumor diagnosis and treatment.

## Introduction

Tumors rank as the second most common cause of mortality globally, posing a notable risk to human health and impacting public health globally [1]. There were nearly 20 million new cases of cancer and 9.7 million deaths from cancer in the year 2022, according to the International Agency for Research on Cancer (IARC) [2,3]. With the aging of the population and changes in environmental factors, the incidence of tumors continues to rise. The primary modalities for treating tumors at present include surgery, radiation, chemotherapy, and molecular-targeted therapy. However, these therapeutic modalities not only are associated with remarkable adverse effects but also suffer from limitations such as drug resistance to radiation and chemotherapy or insensitivity to targeted medications. Due to the inherent limitations of these treatment strategies, as well as the complexity and heterogeneity of tumors, traditional monotherapy is difficult to completely eradicate tumors. These results in frequent relapses and unfavorable patient prognosis. Therefore, there is an urgent need for new technological strategies to overcome these difficulties and provide improved tumor treatment strategies.

With the development of nanotechnology, the role of nanomaterials in biomedical applications has been extensively explored, particularly in relation to the diagnosis and treatment of tumors [4,5]. Because of the enhanced permeability and retention (EPR) effect, the use of nanoparticles as drug transporters can increase drug accumulation at the tumor site while decreasing toxicity to normal tissues. Various types of drug delivery systems, including liposomes, polymeric micelles, carbon nanotubes, and gold nanoparticles, have been used to transport chemotherapeutic medications, targeted therapies, and gene therapeutic agents (such as siRNA). Such approaches can enhance the effectiveness of the treatment and reduce the occurrence of side effects [6,7]. Moreover, nanocarriers can also transport immune checkpoint inhibitors, enhancing the effects of immunotherapy, or can be engineered as nanovaccines that can stimulate the immune system to facilitate antitumor immune responses. Through surface modification, nanoparticles can specifically recognize receptors on the surface of tumor cells, enabling precise drug delivery and enhancing the targeting specificity of the treatment. Smart nanomaterials are able to release medications based on the specific characteristics of the tumor microenvironment (TME), such as the pH and enzyme concentration, or external stimuli, such as magnetic fields and ultrasound [8–14]. Furthermore, some photothermal nanomaterials, such as carbon dots and gold nanorods, can be used as carriers for noninvasive treatments [15]. When exposed to near-infrared (NIR) light, these materials produce locally high temperatures that can destroy tumor cells while causing the least amount of harm to the surrounding healthy tissues [16]. These therapies are often combined with imaging techniques, such as magnetic resonance imaging (MRI), computed tomography (CT), and ultrasound, to facilitate visual monitoring of the treatment process. On the one hand, nanomaterials can enhance imaging contrast and improve diagnostic accuracy. On the other hand, nanoparticles can serve as diagnostic and theranostic integrated platforms, triggering the switch during the diagnosis process to achieve therapeutic effects [4]. Though many nanotechnology-based therapies have demonstrated encouraging outcomes in animal models and early clinical trials, there are still many obstacles to overcome before implementing these technologies in clinical practice. Such challenges include long-term biocompatibility, large-scale production, cost–benefit analysis, and safety assessment.

Manganese (Mn), a vital trace element in the human body, has progressively gained attention in recent years because of its possible connection to cancers. Studies have shown that Mn ions can inhibit the growth of cancer cells [17]. This is because they can affect the TME, disrupt the redox balance in cancer cells, and interfere with the proliferative ability of cancer cells. In the presence of HCO_3_^−^ in the physiological environment, Mn ions perform a Fenton-like reaction with H_2_O_2_ to produce extremely reactive hydroxyl radicals (·OH). They can also catalyze the decomposition of H_2_O_2_, which is abundant in the TME, to produce oxygen (O_2_) at the tumor site, thereby alleviating hypoxia in the TME. Meanwhile, Mn ions can convert the highly expressed glutathione (GSH) in the TME into glutathione disulfide (GSSG), thereby reducing the scavenging effect of GSH on ·OH. In addition, Mn ions possess immunomodulatory functions and can serve as potential immune adjuvants to activate the body’s innate immunity. Consequently, there is a growing interest in the development and investigation of nanomaterials based on Mn. Mn-based nanomaterials are inorganic nanoparticles with Mn as the molecular skeleton. Depending on the form of Mn in the nanoparticles, they can be classified into MnO_2_, Mn_3_O_4_, MnPO_4_, etc., with MnO_2_ being the most common form [18]. The primary techniques for the preparation of Mn-based nanomaterials are hydrothermal synthesis, templating, and sol-gel. As research on the application of Mn-based nanomaterials in tumor diagnosis and treatment progresses, an increasing number of Mn-containing inorganic or organic nanomaterials, such as mesoporous materials, quantum dots, liposomes, metal-organic frameworks (MOFs), and covalent organic frameworks (COFs), are being created [19–23]. The addition of Mn combined with different treatment methods performed by the nanomaterials can reduce the side effects of traditional therapies, such as traditional chemotherapy and radiation therapy (RT). By leveraging the advantages of nanoparticles and enhancing tumor targeting, manganese’s inherent antitumor properties can be harnessed to improve treatment efficacy. Recent studies have demonstrated that 2-dimensional (2D) risedronate-Mn nanobelts can effectively mitigate hypoxia induced by RT. These nanosheets enhance the release of tumor-associated antigens and considerably boost the anticancer immune response [24]. Mn-based nanomaterials also improve the limitations of single therapy for tumors by combination with various therapies, such as chemodynamic, sonodynamic, and immunotherapy, among others, and produce synergistic effects, giving these treatments enhanced antitumor capabilities or accuracy of tumor diagnosis due to Mn’s magnetic responsiveness [25]. A combination of Mn^2+^ and immunotherapy has advanced to phase I clinical trials, demonstrating promising outcomes in the majority of patients with advanced tumors. This indicates that Mn-based nanomaterials possess meaningful potential for clinical translation [17].

**Fig. 1.**
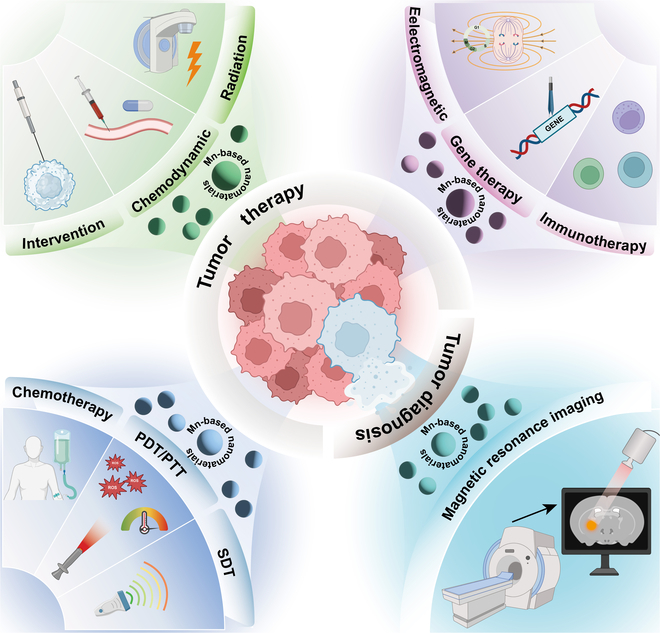
Schematic illustration of applications of Mn-based nanomaterials in tumor diagnosis and therapy. Mn-based nanomaterials boost the effectiveness of conventional tumor therapies such as chemotherapy, radiation, and gene therapy, and synergize with advanced therapeutic strategies including immunotherapy, PTT and PDT therapies, electromagnetic hyperthermia, SDT, chemodynamic therapies, and intervention therapy. Additionally, Mn-based nanomaterials could enhance MRI contrast and support multimodal imaging, improving both treatment outcomes and diagnostic accuracy. MRI, magnetic resonance imaging; PDT, photodynamic therapy; PTT, photothermal therapy; SDT, sonodynamic therapy.

Despite this progress, there remains a scarcity of complete and systematic evaluations of the utilization of various forms of Mn-based nanomaterials in the diagnosis and treatment of tumors. The value of the current work lies in our careful curation of important research findings over the past 10 years and our meticulous and comprehensive synthesis of the most recent developments and treatment approaches involving Mn-based nanomaterials for the diagnosis and treatment of tumors. Firstly, based on the importance of Mn as a trace element in the human body, its physiological functions are introduced. Then, the relevant mechanisms of Mn-based nanomaterials in tumor therapy are summarized. The emphasis of the remainder of this paper is on delineating the integrated utilization of Mn-based nanomaterials alongside diverse diagnostic and therapeutic strategies in the field of oncology. A comprehensive comparison and discussion are provided on the current issues and future development directions of Mn-based nanomaterials in tumor diagnosis and treatment, and the prospects for their future clinical translation and application are discussed. Figure 1 is a schematic illustration of applications of Mn-based nanomaterials in tumor diagnosis and therapy. This comprehensive analysis not only elucidates the complex interactions of manganese in tumor biology but also provides valuable insights into its potential therapeutic applications, highlighting the relevance of Mn-based nanomaterials in contemporary cancer treatment strategies. It contributes remarkably to the existing literature and serves as a valuable resource for researchers and clinicians interested in the intersection of nanotechnology and oncology.

## Physiological Functions of Mn and Mn as a Modulator in Tumor Biology

### Physiological functions of Mn

Mn, an essential trace element, is critical for various biochemical reactions and supports functions related to the nervous system, bone growth, glycometabolism, amino acid, and lipid metabolism [26,27], serving as a cofactor for numerous enzymes, such as oxidoreductases and hydrolases, transferases, and lyases. For instance, Mn^2+^ is a cofactor for superoxide dismutase 2 (SOD2), which is essential for cellular redox balance and protects cells from oxidative stress damage. Also, Mn is preferentially used by most human glycosyltransferases, such as β-1,4-galactosyltransferase 1, participating cellular glycosylation processes by coordinate nucleotide sugars [28]. In astrocyte, a specialized glial cell, glutamine synthetase is one of the most abundant Mn-based proteins, which controls and converts toxic levels of glutamate to the less toxic glutamine to maintain central nervous system homeostasis [29]. Additionally, in other biological interfaces such as host/microbiota, host cells restrict the availability of manganese to combat microbial infections. For instance, host cells can utilize metal transporters such as NRAMP1 to pump manganese out of phagosomes, reducing the pathogen’s access to manganese [30]. In response to host nutritional immunity, microorganisms have evolved various strategies to acquire manganese. For example, some bacteria can secrete high-affinity manganese transport proteins, such as MntH and MntABC, to compete for host-chelated manganese [31]. Mn is absorbed primarily through the diet, with daily requirements set at approximately 1.8 mg for women and 2.3 mg for men [32]. Both a deficiency and an excess of Mn can adversely affect human health. At optimal levels, Mn functions as an antioxidant, aiding detoxification processes via oxidoreductases by catalyzing the conversion of cellular peroxides to hydrogen peroxide [32,33]. However, excessive Mn can lead to neurotoxicity and is associated with neurodegenerative diseases like Alzheimer’s disease and Parkinson’s disease, as well as metabolic disorders such as liver cirrhosis and diabetes mellitus [34,35].

### Molecular mechanisms of Mn in the tumor–immunity cycle

**Fig. 2.**
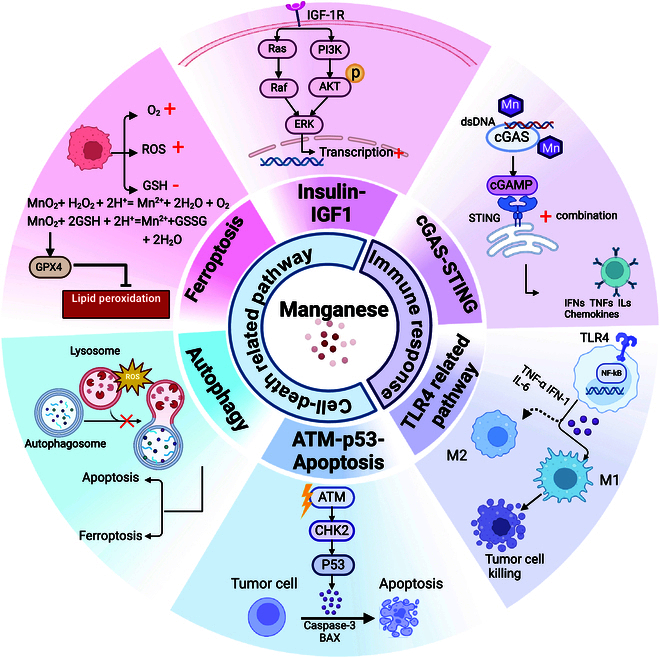
Schematic illustration of molecular mechanisms of Mn in tumor biology. Manganese enhances the body’s antitumor immune responses by cGAS-STING and TLR4 pathways, thereby improving recognition and destruction of tumor cells. Mn influences cell fate via the insulin-IGF1 pathway, the ATM-P53 pathway, autophagy, and the ferroptosis-related pathway, thereby exerting a complex effect on tumor development and progression. cGAS, cyclic GMP-AMP synthase; cGAMP, cyclic GMP-AMP; STING, stimulator of interferon genes; dsDNA, double-stranded DNA; TLR4, toll-like receptor 4; NF-κB, nuclear factor kappa B; IFNs, interferons; TNFα, tumor necrosis factor alpha; ILs, interleukins; IGF-1R, insulin-like growth factor 1 receptor; PI3K, phosphoinositide 3-kinases; AKT, protein kinase B; ERK, extracellular signal-regulated kinase; RAF, rapidly accelerated fibrosarcoma; GPX4, glutathione peroxidase 4; ROS, reactive oxygen species; ATM, ataxia telangiectasia mutated; p53, tumor protein p53; Bax, BCL-2-associated X protein; CHK2, checkpoint kinase 2. The figure was created by BioRender.

Recent studies have further elucidated the complex role of Mn in tumor biology, revealing how Mn influences tumor cell metabolism, growth, and apoptosis through specific signaling pathways and molecular mechanisms. These insights underscore the critical need for precise regulation of Mn homeostasis to maintain the overall health of the body and mitigate cancer progression. The following sections will delve into the mechanism by which Mn regulates intracellular physiological and pathological processes, with a particular focus on its role in tumor biology. Figure 2 is a schematic illustration of molecular mechanisms of Mn in tumor biology.

#### Mn as a modulator of the immune response

##### Enhancing the immune response through the cGAS-STING pathway

Mn exists in various oxidation states, but Mn^2+^ is the most stable and biologically major form, crucial for multiple physiological processes. Recent research highlights the pronounced impact of Mn^2+^ on the cyclic GMP-AMP synthase (cGAS)-stimulator of interferon (IFN) genes (STING) pathway, which is essential for immune responses to infections and tumors.

The cGAS-STING pathway, activated by the detection of cytoplasmic double-strand DNA (dsDNA) from pathogens or cellular damage, plays a vital role in antitumor biology. It can kill tumors and inhibit tumor metastasis, creating a tumor-suppressive microenvironment [36,37]. cGAS is an enzyme that detects dsDNA and synthesizes the secondary messenger cGAMP. Mn^2+^ can be released from cellular organelles such as the mitochondria and Golgi apparatus, particularly during viral infections, leading to a substantial increase in the cytosolic Mn^2+^ concentration. Mn^2+^ improves the sensitivity of cGAS to dsDNA, enabling it to produce cGAMP even at low dsDNA levels. Moreover, Mn^2+^ increases the binding affinity between cGAMP and STING, amplifying the activation of STING. This hyperactivation lowers the detection threshold for tumor-derived DNA, leading to a robust innate immune sensing of tumors. Moreover, Mn^2+^ can also activate cGAS independently of dsDNA and can accelerate the overall catalytic activity of cGAS, exhibiting broad-spectrum antiviral activity [38]. In a cGAS-STING-dependent way, Mn^2+^ was found to significantly promote antigen-presenting cell maturation and antigen presentation, augment CD8^+^ T cell and natural killer (NK) cell activation, and increase the number of CD8^+^ T cells, thereby significantly boosting antitumor immunotherapies in various mouse models [17]. In vivo, Mn-based chemical compounds have been shown to block lung metastases in wild-type but not *Sting1*^−/−^ mice, suggesting that Mn could serve as an immune potentiator and a potent antitumor vaccine [39].

##### Activating immune responses via TLR4 signaling

Toll-like receptor 4 (TLR4) is a key pattern recognition receptor on the cell membrane that detects lipopolysaccharide (LPS) through a complex involving CD14 and myeloid differentiation factor 2. LPS binding activates TLR4, leading to its dimerization and the recruitment of the adaptor proteins MyD88 and TRIF. This activation triggers 2 main pathways: the MyD88-dependent pathway, which activates interleukin-1 receptor associated kinases and IκB kinase ε, resulting in nuclear factor kappa-B (NF-κB) activation and pro-inflammatory cytokine expression, and the Toll/IL-1 receptor domain-containing adaptor (TRIF)-dependent pathway, which activates IFN regulatory factor 3 (IRF3) and IFN regulatory factor 7 (IRF7), leading to type I IFN production. Dysregulation of TLR4 signaling is linked to heart failure and tumor progression, making it a target for therapies aimed at reducing inflammation and inhibiting tumors.

Mn plays a multifaceted role in modulating TLR4 signaling, impacting both immune responses and neuroinflammation. Mn^2+^ exposure has been shown to activate TLR4-mediated signaling cascades, which play an important role in the inflammatory responses of macrophages [40]. For instance, Mn@bovine serum albumin (BSA) nanocomplexes were found to enhance pro-inflammatory responses and macrophage activation, demonstrating the potential of Mn as an adjuvant in cancer immunotherapy and its impact on macrophage-mediated innate immunity [41]. Additionally, with Mn overexposure in Parkinson’s disease rat models, neuroinflammatory biomarkers, such as tumor necrosis factor-α (TNF-α), TLR4, nucleotide-binding oligomerization domain, leucine-rich repeat and pyrin domain-containing 3 (NLRP3), NF-κB, Caspase-1, and interleukin-1β (IL-1β), were increased significantly, suggesting that Mn is involved in neurodegenerative processes through inflammatory mechanisms, such as TLR4/NLRP3/NF-κB [42]. Thus, the role of Mn in TLR4 signaling highlights its substantial impact on both systemic and neuroinflammatory responses.

#### Mn as a modulator of cell fate

##### Promoting tumor cell death through ferroptosis

Ferroptosis is an iron-dependent form of cell death characterized by oxidative stress and lipid peroxidation. It involves the production of reactive oxygen species (ROS) through 2 primary mechanisms: the Fenton reaction, where iron reacts with hydrogen peroxide to generate ROS, and the lipid peroxidation of polyunsaturated fatty acids on cell membranes, leading to toxic lipid radicals [43]. The key regulator of ferroptosis is glutathione peroxidase 4 (GPX4), which neutralizes lipid peroxides into nontoxic lipid alcohols. This process relies on GSH supplied by the cystine/glutamic acid transport system, particularly the Xc-system components recombinant solute carrier family 7, member 11 and recombinant solute carrier family 3, member 2 [44].

Recent research has revealed that Mn^2+^ is an essential modulator of ferroptosis. Mn^2+^ can enhance ROS production by catalyzing a Fenton-like reaction, where it reacts with hydrogen peroxide to generate harmful hydroxyl radicals (·OH), thus amplifying ferroptosis in tumor cells [44]. Additionally, Mn^2+^ depletes GSH levels, inhibiting GPX4 activity and further promoting ferroptosis. The role of Mn^2+^ extends to influencing dihydroorotate dehydrogenase, a key molecule in ferroptosis [44,45].

The potential of Mn in cancer treatment is supported by its ability to induce ferroptosis and inhibit tumor cell proliferation. Studies show that Mn can be effectively utilized in various therapeutic modalities, including chemical therapy, RT, photodynamic therapy (PDT), and others [46]. Additionally, Mn-induced ferroptosis has been linked to the cGAS-STING pathway, which enhances its efficacy as a therapeutic agent in cancer treatment [47,48].

##### Triggering apoptosis via the ATM-P53 pathway

Recent research has revealed the important role of Mn in apoptosis and the modulation of the ataxia telangiectasia mutated protein (ATM)-tumor suppressor protein 53 (p53) signaling pathway, which is essential for cellular responses to stress and DNA damage. Mn affects apoptosis through its impact on forkhead transcription factors acetylation by sirtuin (SIRT), leading to the up-regulation of apoptosis-related molecules such as p53 [49]. Specifically, Mn-ZnO_2_ nanoparticles have been shown to induce ubiquitination-mediated degradation of mutant p53 (Mutp53) while activating WTp53 via the ATM-p53-BCL-2-associated X protein (Bax) pathway, thereby enhancing antitumor efficacy [50]. Additionally, Mn exposure notably affects p53 phosphorylation at serine 15, a process dependent on ATM kinase activation. This Mn-dependent activation of p53 is notably diminished in Huntington’s disease models, indicating a selective alteration in Mn handling and ATM activation in this condition [51]. Mn-induced neurotoxicity, along with its effects on the ATM-p53 pathway, is influenced by SIRT1 modulation and mitochondrial dysfunction, highlighting the complex interplay between Mn, ATM kinase activity, and p53 signaling [49]. The impact of Mn on the ATM-p53 pathway underscores its critical role in the regulation of cellular stress responses and various disease processes.

##### Modulating autophagy and apoptosis

Autophagy, an essential cellular process for the degradation and recycling of intracellular components, maintains cellular homeostasis under stress conditions such as endoplasmic reticulum stress, starvation, and hypoxia. There are 3 main types of autophagy: macroautophagy, microautophagy, and chaperone-mediated autophagy, each crucial for regulating intracellular protein levels and ensuring cellular function. Mn plays a multifaceted role in these processes. Mn compounds, such as Adpa-Mn, influence both autophagy and apoptosis. On one hand, Mn induces apoptosis in cancer cells by triggering key apoptotic markers, including chromatin condensation and the activation of caspases (caspase-3, -7, and -9), while causing mitochondrial damage characterized by increased ROS and disrupted membrane potentials [49]. On the other hand, Mn stimulates autophagy by up-regulating proteins such as microtubule-associated protein 1 light chain 3 and Beclin-1, essential for cellular degradation and recycling [52]. Mn also acts as a cofactor for the target of rapamycin complex 1 protein kinase, initiating autophagy, mitophagy, and mitochondrion-to-nucleus retrograde signaling [53]. This dual role—enhancing both autophagy and apoptosis—depends on the cellular context. In cancer cells, Mn-induced autophagy can amplify its apoptotic effects, presenting a potential therapeutic strategy. However, in other contexts, Mn-induced autophagy might protect cells from apoptosis, highlighting the complex interplay of these processes and the need for a nuanced understanding of the role of Mn in various pathological settings [54].

##### Modulation of the insulin/IGF-1 signaling pathway

Mn plays a critical role in regulating the insulin/insulin-like growth factor 1 (IGF-1) signaling pathway, which is essential for controlling cellular growth, metabolism, and proliferation. This pathway, mediated by receptor tyrosine kinases such as IGF-1R and IGF-2R, responds to ligands like insulin, IGF-1, and IGF-2. Upon activation, these receptors phosphorylate tyrosine residues on substrates such as insulin receptor substrate and Src homology 2 domain containing, leading to the recruitment and activation of downstream signaling molecules, including phosphatidylinositol 3-kinase (PI3K), growth factor receptor-bound protein 2, and son of sevenless, which, in turn, activate key pathways such as PI3K/protein kinase B (AKT), mitogen-activated protein kinase (MAPK), and signal transducer and activator of transcription. Mn acts as a cofactor to enhance the activation of these insulin/IGF-1 receptors, thereby boosting the phosphorylation of insulin-like growth factor 1 receptor (IR/IGFR) and downstream kinases such as AKT. Mn’s modulation of the insulin/IGF-1 signaling pathway highlights its vital role in the regulation of cellular functions and its impact on various disease states [55,56]. In particular, in tumors, Mn was found to promote adhesion to fibronectin through very late antigen-4 activation and stimulate the MAPK pathway, supporting multiple myeloma cell growth and proliferation [57]. The influence of Mn on IGF-1 signaling is distinct from that of other divalent cations, highlighting its unique role in the modulation of this pathway. In contrast, Mn-based Prussian blue nanoparticles have been shown to inhibit tumor proliferation and migration in pancreatic cancer [58]. Therefore, the above research indicates that there is uncertainty as to the regulatory of Mn in tumors through the MAPK pathway, which is downstream of insulin/IGF-1 signaling.

In conclusion, Mn can act as an activator of innate immunity to regulate immune response and participate in the regulation of a variety of cell death-related signaling pathways to directly affect cell fate. Therefore, Mn-based antitumor therapy may have a long-term development prospect. Solid tumors have a highly heterogeneous TME, which consists of tumor cells, immune cells, fibroblasts, and other cell types, as well as noncellular components such as extracellular matrix (ECM) and growth factors. The interactions between these components determine the fate of the tumor, including its growth, progression, metastasis, and response to treatment. Among them, the innate immune cells in the TME are very important for the immune surveillance of the tumor. Manganese can enhance the antitumor effect of macrophages by promoting their M1-type polarization [59]. Besides, manganese can activate dendritic cells (DCs) to enhance their antigen-presenting ability, and then promote the recognition and killing of tumor by T cells [60]. In a manganese-deficient mouse model, the rate of tumor growth is significantly increased and there is a higher tendency for tumor metastasis, whereas the exogenous addition of Mn^2+^ effectively stimulates host immune cells, promotes CD8^+^ cells’ invasion within tumor tissue, and enhances their specific killing of tumor cells [17]. The immunosuppressive state of the TME is a major challenge in cancer treatment, and manganese, as a potential immune adjuvant, can inhibit tumor development by modulating the tumor immune microenvironment. By promoting the function of innate immune cells, manganese may effectively relieve the immune escape mechanism of tumors, thereby enhancing the surveillance and clearance of tumors by the immune system. Therefore, the development of suitable delivery systems to target Mn delivery into the TME is a highly feasible treatment plan.

## Application of Mn-Based Nanomaterials in Tumor Diagnosis and Therapy

In clinical practice, surgery is the most direct treatment method for tumors that are detected early and are in an appropriate location, in particular, for benign and partially malignant tumors. For unresectable tumors and postoperative adjuvant therapy, chemotherapy, RT, and other new treatment modalities, such as targeted therapy, immunotherapy, etc., are the main approaches used in clinical practice [61–63]. The combination of Mn-based nanoparticles with traditional or novel therapeutic modalities offers new strategies for the treatment of tumors. Mn-based nanoparticles retain the antitumor function and advantages of nanoparticles, while the addition of Mn enhances the antitumor effect through synergistic actions [21,22,25,64]. Tumor diagnosis usually occurs clinically through blood tests and imaging tests, such as ultrasound, CT, and MRI. The advantage of Mn-based nanoparticles in tumor diagnosis is that the magnetic properties of Mn itself can enhance the imaging contrast of MRI or achieve multimodal imaging with fluorescence, ultrasound, or CT [65–67]. The following section introduces the application of Mn-based nanomaterials in tumor diagnosis and treatment.

### Application of Mn-based nanomaterials in tumor therapy

With progress in our understanding of the mechanisms of tumor development, in addition to the traditional treatment methods such as surgery, chemotherapy, and RT, an increasing number of treatment strategies have been applied to tumor therapy, including immunotherapy, gene therapy, photothermal therapy (PTT), electromagnetic therapy, ultrasound therapy, chemical kinetic therapy, and interventional therapy. The synergistic effect between Mn-based nanomaterials and these treatment modalities has been shown to improve the tumor treatment effect.

#### Combination with chemotherapy

Chemotherapy currently remains one of the standard treatments provided after surgical resection of tumors. However, the nonselective killing of cells by chemotherapeutic drugs leads to strong side effects, which seriously affect the quality of life of patients. At the same time, the problem of drug resistance due to the long-term use of chemotherapy drugs remains an urgent problem to be solved in the field of tumor treatment. The combined application of Mn-based nanomaterials and chemotherapy drugs mainly involves the use of these nanomaterials as carriers of chemotherapy drugs. On the one hand, due to the particle size of nanomaterials, the passive targeting of drugs is realized through the EPR effect, while the antibodies corresponding to tumor surface antigens modified on the surface of nanomaterials can more accurately actively target tumors. In addition, some pH-responsive, photo-responsive, electromagnetic-responsive, redox-responsive, and other responsive Mn-based nanomaterials can achieve the controlled release of chemotherapy drugs, greatly reducing the toxicity and side effects of chemotherapy drugs. In these nanoparticles, Mn primarily exerts its inherent antitumor properties, synergizing with chemotherapy to enhance the antitumor capabilities of the nanoparticles.

Zhang et al. [68] used the thin-film hydration method to design a liposomal nanomaterial MnO_2_-PDA@Lipo/Geb@Beb that is responsive to the microenvironment pH and GSH (Fig. S1A). The authors investigated its effect on non-small-cell lung cancer and its biological safety through in vitro and in vivo experiments. After surface hydrophilic modification of MnO_2_ nanorods by dopamine hydrochloride, the phospholipid vesicles composed of 1,2-dioleacyl-sn-glycero-3-phosphoethanolamine (DOPE) modified by MnO_2_-PDA enclosed the chemotherapeutic drugs gefitinib (Geb) and bevacizumab (Beb). The particle size of the final composite material was around 160 nm. The in vitro release kinetics of Geb and Beb from MnO_2_-PDA@Lipo@Geb@Beb were examined in phosphate-buffered saline (PBS) buffer at pH 5.0 and 7.4. The results demonstrated that, due to the pH sensitivity of DOPE and the pronounced reductive properties of nano MnO_2_, the cumulative release rates of Geb and Beb reached 70% at pH 5.0 after 27 h, surpassing the 20% observed at pH 7.4. In comparison to Geb alone, MnO_2_-PDA@Lipo@Geb and MnO_2_-PDA@Lipo@Geb@Beb exhibited more pronounced inhibitory effects on cancer cells, with the apoptotic ratios of A549 cells being 14.64%, 16.01%, and 22.40%, respectively, at a concentration of 1 μg/ml over 24 h. While all groups exhibited some toxicity to 16 HBE normal bronchial epithelial cells, the application of the MnO_2_-PDA@Lipo composite material mitigated the detrimental effects of these medicines on normal cells. In a BALB/c nude mouse model injected with A549 cells, the tumor inhibition rates for the Geb, MnO_2_-PDA@Lipo@Geb, and MnO_2_-PDA@Lipo@Geb@Beb groups were 32.80%, 53.56%, and 78.54%, respectively. In the assessment of drugs that induce systemic toxicity in vivo, no discernible tissue abnormalities were noted in the normal organs of mice within the nanodrug groups.

Photo-responsive biomaterials, which undergo changes in their physical and chemical properties under light, have been widely used in biomedical fields, such as in controlled drug release systems. Glucose transporter 1 (GLUT-1) is a membrane protein that is highly expressed in most tumors. Yang et al. [69] described an aptamer-modified photo-responsive nanoparticle targeting GLUT-1. The Mn-D@B nanoparticle, with Mn-DOX coated by BSA, was modified with polyethylene glycol–ethanoic acid (PEG-FA), which could further coordinate with Fe^3+^ forming the nanoparticle Mn-D@BPFe (Fig. S1B). Mn-D@BPFe bound to the linker sequence and matched with the GLUT-1 recognition aptamer, resulting in a nanoreactor (Mn-D@BPFe-A). The dimensions of the nanoreactor ranged from 20 to 40 nm. Under the irradiation of red light-emitting diode light (630 to 700 nm, 4 W), water was catalyzed to create O_2_ by Mn-D@BPFe nanoparticles, thereby confirming its photo-responsive nature in neutral conditions. Upon photoexcitation at 498 nm, HepG-2 cells treated with Mn-D@BPFe-A exhibited greater fluorescence intensity compared to those treated with Mn-D@BPFe. The in vitro tetrazolium salt test (MTT) cytotoxicity experiment demonstrated that Mn-D@BPFe-A exhibited greater cytotoxicity toward HepG-2 cells in a dose-dependent manner, with an IC_50_ value of 18.62 μg/ml under irradiation and 51.24 μg/ml in the absence of irradiation. The synergistic anticancer efficacy of the nanoreactor was evaluated in HepG-2 tumor-bearing nude mice, revealing that Mn-D@BPFe-A exhibited superior antitumor effects with minimal toxicity and great biocompatibility. The results also demonstrated that the Mn-D@BPFe-A nanoparticle substantially altered the shape of tumor tissues due to light-induced cytotoxicity.

Due to the excessive concentration of GSH in the TME and the variable valence state of Mn, Mn^2+^ can act as a redox-responsive agent. Redox-responsive MnO_2_-Pt (IV) nanostructures (Fig. S1C) were synthesized through a one-pot facile ultrasonication reaction from KMnO_4_ and a functionalized Pt (IV) prodrug complex [70]. In an environment characterized by high GSH, Mn^4+^ is reduced to Mn^2+^. Mn^2+^ has stronger para-magnetism than Mn^4+^ and has a greater influence on the imaging effect of MR signals. The Pt (IV) complex can reduce to Pt (II) to kill tumor cells. The size of the MnO_2_-Pt (IV) nanostructures was 135 ± 24 nm, and they displayed a negative surface charge in aqueous solution at neutral pH (−53.0 ± 0.4 mV). With reducing agents (ascorbic acid), 42.6% and 71.4% of Mn was liberated from the nanoparticles at the conclusion of the experiment in pH 7.4 and 5.5 buffer, respectively. To assess the efficacy of these nanostructures as therapeutic agents, in vitro toxicity studies were conducted using 2D and 3D cell models of A549 human non-small-cell lung carcinoma cells. The toxicity of the nanostructures (IC_50_ = 100.0 μM) was significantly greater than that of the precursor Pt (IV) prodrug (IC_50_ = 420.5 μM) in the 2D cell models, with analogous results observed in the 3D cell models. This suggests a potential synergistic or cooperative interaction between Mn and Pt.

Due to its paramagnetism, Mn can also be used as a magneto-thermally responsive material. It also exhibits synergistic effects with other magneto-thermally responsive materials on the release of drugs in tumors. Li et al.[71] synthesized magneto-thermally responsive nanocarriers/doxorubicin (MTRN/DOX) composed of Mn-Zn-Fe magnetic nanoparticles (MZF), amphiphilic and thermosensitive copolymer drug carriers, and DOX through self-assembly. CD147-MTRN/DOX was formed with MTRN/DOX and the monoclonal antibody that specifically binds to CD147 protein, due to the high expression of CD147 in hepatoma cells (Fig. S1D). Results of in vitro experiments, including transmission electron microscopy (TEM), inductively coupled plasma-atomic emission spectrometry, flow cytometry, and fluorescence staining, revealed that CD147-MTRN/DOX exhibited greater targeting of liver tumor cells of Huh-7 than normal liver cells of L-02, as compared with MTRN/DOX, and induced a higher DOX release rate in cells. The in vivo enrichment of nanoparticles on tumors via tail vein injection was consistent with the in vitro results. Under the application of an alternating magnetic field (AMF) for 20 min, CD147-MTRN/DOX had better magneto-thermal effects in vivo, with the tumor temperature continuously elevated to 53.2 °C, while the tumor temperature was 43 °C in the MTRN/DOX group. Furthermore, investigation of the nude mouse cancer model demonstrated that CD147-MTRN/DOX exhibited excellent biocompatibility with minimal toxicity, as well as direct targeted aggregation to tumor facilitated by the CD147 monoclonal antibody and the synergistic effects of thermotherapy and chemotherapy. Overall, the data demonstrated that CD147-MTRN/DOX has substantial anticancer therapeutic efficacy.

#### Combination with RT

RT is a common adjuvant in the treatment of tumors. It primarily involves the use of ionizing radiation to induce DNA damage to kill cancer cells. One of the major challenges of RT is the resistance of tumors to RT, which will greatly affect its therapeutic effect. This is because RT primarily stimulates tissues or cells to produce ROS through ionizing radiation, inducing a tumor killing effect, and the hypoxic microenvironment of tumors will restrict the effect of RT. Mn-based nanomaterials can regulate the TME and relieve the oxygen state of tumors and, thus, when combined with RT, can improve the tumor-killing ability. Furthermore, RT enhances immune responses associated with tumors. Furthermore, RT has been documented to elicit immunogenic cell death (ICD), which is the immunogenic component present in all types of cell death [72]. Due to its effects on the TME, Mn-based nanomaterials can alleviate the hypoxia caused by RT, thereby enhancing its sensitivity. The immunomodulatory properties of manganese can also synergize with other treatments to kill tumor cells.

Liu et al. [73] described biomineralized MnO_2_ nanoparticles (Bio-MnO_2_ nanoparticles) prepared by a mild enzymatic reaction. Recombinant multicopper oxidase MnxEFG was expressed and purified from *Escherichia coli*. MnxEFG was added to the biomineralization buffer and Bio-MnO_2_ nanoparticles were obtained via a biomineralization process. The average hydrodynamic diameter of the nanoparticles was 101 nm, as measured by dynamic light scattering (DLS), with a polydispersity value of 0.122. Up to 25 μg/ml of Bio-MnO_2_ nanoparticles was used, with no significant cytotoxicity. Cy3-labeled Bio-MnO_2_ nanoparticles were then utilized to verify their effective absorption capability in A549 and PC9 cells. The introduction of 25 μg/ml Bio-MnO_2_ nanoparticles resulted in a reduction in H_2_O_2_ levels of roughly 30% to 50% as compared to untreated cells. Furthermore, ionizing radiation-induced H_2_O_2_ was inhibited by over 50% with the use of Bio-MnO_2_ nanoparticles. The radio-sensitizing efficacy of Bio-MnO_2_ nanoparticles in the A549, PC9, and H520 cell lines was confirmed using colony formation assays, immunofluorescence for apoptosis-related Ki-67, and flow cytometry for cell apoptosis assessment. The combination of Bio-MnO_2_ nanoparticles with radiation-augmented DNA damage in non-small-cell lung cancer cells heightened the activation of the cGAS/STING signaling pathway and triggered ICD. Tumor-bearing mouse models were then established through subcutaneous injection of 1×10^7^ LLC cells into C57BL/6 mice to assess the in vivo antitumor efficacy of radiation and Bio-MnO_2_ nanoparticles. The Bio-MnO_2_ nanoparticle-mediated radiotherapeutic intervention exhibited the most pronounced effect in suppressing rapid tumor development and eliciting immunological responses in vivo, wherein cytotoxic T cells were activated.

Recent research has indicated that ferroptosis plays a crucial role in the progress of RT and the therapeutic efficiency of RT can be effectively improved by increasing ferroptosis [74,75]. However, the cytoplasm pH value of tumor cells remains an intrinsic challenge for efficient Fenton/Fenton-like reaction-based ferroptosis induction. Zheng et al. [76] engineered TME-activated PEGylated hollow mesoporous organosilica nanotheranostic (HMON)-GOx@MnO_2_ nanoparticles (HGMP nanoparticles). Glucose oxidase (GOx) was conjugated to amino-modified HMON by acylamide condensation. Thereafter, the MnO_2_ shell was applied in situ to the surface of the synthesized HMON-GOx nanoparticles (Fig. S2). The HMON-GOx@MnO_2_ nanoparticles exhibited a mesoporous architecture with an average diameter of 69.07 ± 4.10 nm and a pore diameter of around 1.9 nm. The ultraviolet–visible (UV–Vis) spectroscopy absorption spectra confirmed that GOx in HGMP nanoparticles accelerated the oxidation of glucose, resulting in the production of H_2_O_2_, which then interacted with Mn^2+^ to generate additional ROS. In vitro studies involving normal mouse 3T3-E1 cells and mouse breast cancer 4T1 cells demonstrated that the viability of MC3T3-E1 cells exceeded 70% even at a high concentration of HMP nanoparticles ([Mn]: 4.5 μg/ml) over 24 h. Conversely, at the same Mn concentration, the cytotoxicity of HGMP nanoparticles significantly increased, reaching 77%, in 4T1 cells pretreated with proton pump inhibitors (PPIs); this was facilitated by an enhanced Fenton reaction to induce ferroptosis. These findings suggest that PPI and GOx may enhance RT sensitization in 4T1 cells or 4T1 tumor-bearing mice through ferroptosis produced by HGMP nanoparticles.

#### Combination with PTT/PDT

PTT/PDT is a novel noninvasive tumor treatment method that involves the accumulation of a photothermal agent/photosensitizer in the form of nanomaterials and the use of an external light source (usually NIR) to kill tumors. PTT kills tumors by increasing the local temperature through the photothermal effect, while PDT kills tumors by activating the photosensitizer to react with oxygen to produce ROS. As emerging treatments, PTT and PDT have low toxic side effects and high selectivity for tumors, and can be combined with other treatments such as chemotherapy and RT. Mn-based nanomaterials can sometimes serve as photosensitizers and increase the photothermal conversion efficiency of nanomaterials. Mn in nanomaterials can regulate the TME, generating ROS to enhance the antitumor ability. Furthermore, functional nanomaterials often improve the targeting of photosensitizers. Among them, MnOx, such as MnO_2_ and Mn_3_O_4_, are most commonly employed in Mn-based nanomaterials.

**Fig. 3.**
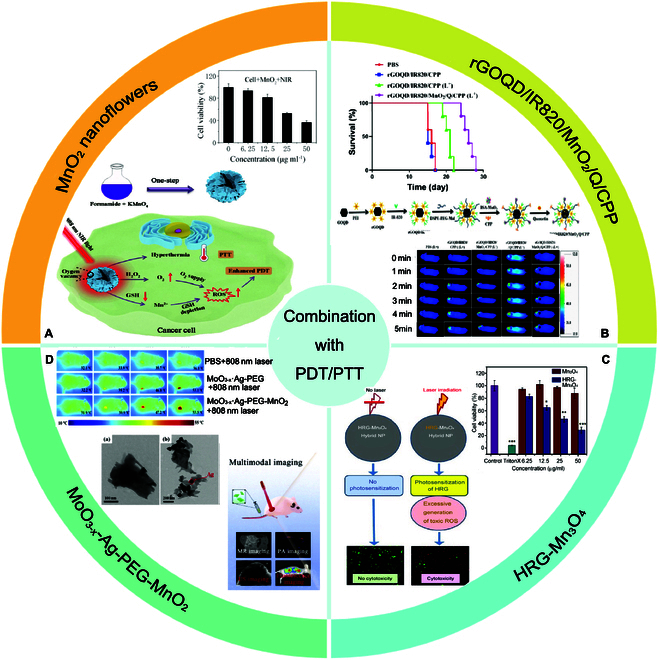
Mn-based nanomaterials combined with photothermal therapy/photodynamic therapy in tumor therapy. (A) MnO_2_ nanoflowers; (B) rGOQD/IR820/MnO_2_/Q/CPP; (C) HRG-Mn_3_O_4_; (D) MoO_3-x_-Ag-PEG-MnO_2_. Panel (A) is adapted with permission from Ref. [77], copyright 2021 *Photochemical & Photobiological Sciences*. Panel (B) is adapted with permission from Ref. [80], copyright 2024 *ACS Applied Materials & Interfaces*. Panel (C) is adapted with permission from Ref. [83], copyright 2023 *Frontiers in Bioscience-Landmark*. Panel (D) is adapted with permission from Ref. [86], copyright 2021 *Journal of Controlled Release*.

Chen et al. [77] successfully developed a nanostructured NIR light-responsive MnO_2_ with a high photothermal conversion capability. MnO_2_ nanoflowers synthesized by KMnO_4_ and methanamide were linked with 3′,6′-dihydroxy-5-isothiocyanato-3H-spiro[isobenzofuran-1,9′-xanthen]-3-one (FITC). The MnO_2_ nanoflowers (Fig. 3A) possessed a large surface area (about 156 m^2^ g^−1^) and macroporosity structure (35 nm). The optical properties of the nanoflowers were analyzed, and the broad absorption of MnO_2_ in the Vis–NIR range suggested that MnO_2_ can be used as a PTT agent. The photothermal conversion efficiency of the MnO_2_ nanoflowers was 30%, which was higher than that reported for Cu_2-x_S (11%)[78], MoO_3-x_ (26%)[79] and black phosphorus quantum dots (28%). As a photocatalyst for PDT application, MnO_2_ nanoflowers exhibited high photostability. In vitro, more than 60% of HeLa cells incubated with 50 μg/ml of MnO_2_ were killed under 808-nm laser irradiation, while most cells survived without laser irradiation. Importantly, most MnO_2_ nanoflowers were significantly collapsed after 24 h. In particular, the framework of MnO_2_ was almost completely degraded after 70 h in a simulated TME with GSH and H_2_O_2_. These findings demonstrate the biosafety of the material in the body.

Besides the optical performance of MnO_2_ itself, MnO_2_ nanomaterials can combine with other photothermal agents or photosensitizers, offering improved optical performance. Reduced graphene oxide quantum dots (rGOQD) are a cytocompatible photothermal agent that was covalently bound with the photosensitizer IR820 by Dash et al. [80] (Fig. 3B). To improve the suspension stability, rGOQD/IR820 was PEGylated for covalent binding with MnO_2_/BSA and the cell-penetrating peptide (CPP) ligand to synthesize rGOQD/IR820/MnO_2_/CPP. The photothermal conversion efficiency (η) remained at almost 46.0% for rGOQD/IR820/MnO_2_/CPP, which was slightly lower than that reported for pheophorbide a-dityrosine nanofibers (48.0%) [81] and peptide-porphyrin photothermal nanodots (54.2%)[82]. MnO_2_ in rGOQD/IR820/MnO_2_/CPP mediated ROS and oxygen gas generation. Quercetin was loaded by π−π interaction, and rGOQD/IR820/MnO_2_/Q/CPP exhibited a strengthened antitumor ability by suppressing the expression of stress-induced HSP70. At the maximum dose assessed (100 μg/ml), cell viability exceeded 90% for both cell lines. The cell apoptosis rate of rGOQD/IR820/MnO_2_/Q/CPP reached nearly 89%, which was higher than the other groups. The in vivo tumor-bearing mouse model was then studied. CPP in rGOQD/IR820/MnO_2_/Q/CPP facilitated the ability of the material to cross the blood–brain barrier (BBB) and accumulate in intracranial U87 tumors of nude mice, demonstrating its targeting ability. The photothermal effect of rGOQD/IR820/MnO_2_/Q/CPP was increased from 34 to 51 °C in 5 min, compared with from 34 to 39 °C for PBS. The median survival time of the tumor-bearing mice after injection of rGOQD/IR820/MnO_2_/Q/CPP was increased to 26 days.

Due to the unique optical, physicochemical, and biomedical properties of graphene-based nanomaterials, Khan et al. [83] used manganese oxide (Mn_3_O_4_) and highly reduced graphene oxide (HRG) to synthesize hybrid nanoparticles (HRG-Mn_3_O_4_) by the milling process (Fig. 3C). The newly synthesized nanoparticles were characterized by TEM, energy-dispersive x-ray spectroscopy, UV–Vis spectroscopy, Fourier transform infrared spectroscopy, thermogravimetry, and x-ray diffraction analyses. The shape of the hybrid nanoparticles was round with an average diameter of 12 ± 2.21 nm. The cytotoxicity of the nanomaterials was analyzed using MTT assays; more than 98% of the A549 cells survived even after exposure to a high concentration (100 μg/ml), demonstrating the material’s biocompatibility. Importantly, after laser irradiation of 670 nm for 5 min, the HRG-Mn_3_O_4_ nanoparticles induced significant and concentration-dependent cellular damage, as compared to PBS and Mn_3_O_4_. Moreover, the HRG-Mn_3_O_4_ nanoparticles increased the intracellular ROS generation in A549 cells in the presence of laser irradiation. These findings indicate that these nanoparticles are biocompatible and, after exposure to laser light at a specific wavelength, can induce massive cellular damage, highlighting their significant potential for PDT.

According to a previous report, plasmonic MoO_3-x_ has a strong NIR absorption and the nanocomposite of MoO_3-x_ nanosheets and Ag nanocubes (MoO_3-x_-Ag) can absorb NIR region light efficiently, not only converting light to heat but also generating ROS [84,85]. In this study, the nanocubes of MoO_3-x_-Ag were modified with PEG-NH_2_ and MnO_2_ nanoparticles of about 15 nm in size [86] (Fig. 3D). The MnO_2_ nanoparticles were well dispersed on the surface of the MoO_3-x_ nanosheets with good stability. The diameter of MoO_3-x_-Ag-PEG-MnO_2_ was 310 nm. The photothermal conversion efficiency of MoO_3-x_-Ag-PEG-MnO_2_ under an 808-nm laser was 37%, which was higher than other previously reported agents [87,88]. Further, under 808-nm laser stimulation, MnO_2_ of MoO_3-x_-Ag-PEG-MnO_2_ generated O_2_ or ROS from H_2_O_2_ and depleted GSH, relieving the hypoxia of the TME and improving the PDT effect. Moreover, MoO_3-x_-AgPEG-MnO_2_ exhibited good blood compatibility and biodegradation behavior in vitro and in vivo. In the in vitro HeLa and MCF-7 cell models, an obvious decrease in cell viability was observed in the MoO_3-x_-Ag-PEG +808 nm laser group due to the photothermal and PDT effects of MoO_3-x_-Ag-PEG. In the nude mouse model, after irradiation with an 808-nm laser for 5 min, the tumor temperature increased to 53.5 °C, demonstrating that MoO_3-x_-Ag-PEG-MnO_2_ could serve as an ideal photothermal agent for cancer PTT. Moreover, the tumor weight was significantly inhibited. Finally, hematoxylin and eosin staining of tumor slices indicated that MoO_3-x_-Ag-PEG-MnO_2_ combined with an 808-nm laser had the most significant tumor-suppressive impact relative to the other groups.

#### Combination with electromagnetic therapy

Tumor electromagnetic therapy is a therapeutic method that uses alternating electric fields of specific frequencies to interfere with the mitosis of tumor cells and thereby inhibit tumor growth. This method reduces the trauma and side effects on the patient’s body due to its noninvasive nature. However, its high cost and individual differences limit its application in clinical practice. Currently, several nations have sanctioned its use for the treatment of particular malignancies, including glioblastoma. Nevertheless, further clinical research is needed to validate its safety and efficacy. Mn exhibits unique magnetic responsiveness in nanoparticles, which, on one hand, enhances the ability of drug release targeting tumors and, on the other hand, endows them with diagnostic functionality. We further discuss this in the “Application of Mn-based nanomaterials in tumor diagnosis” section.

Extremely low-frequency electromagnetic field (ELF-EMF) is a noninvasive and nonionizing method that also exhibits nonthermal effects on cells and tissues [89,90]. Schiff bases are an important group of organic compounds with a wide variety of biological properties, including antitumor abilities [91]. Therefore, Yadamani et al. [92] designed an Mn(II) complex of the N,N0-dipyridoxyl(1,2-diaminobenzene) Schiff base, which was used for the treatment of breast tumors. The cytoplasm and cell cycle of the breast tumor cell line TUBO were gradually destroyed with an Mn(II) complex concentration ranging from 31.2 to 125 μg/ml. Under an ELF-EMF of 50 Hz, apoptosis of transplanted breast cancer tissue in mice treated with the Mn(II) complex was increased, demonstrating its superior inhibition ability.

Under AMFs, magnetic nanomaterials can be coupled to chemotherapeutic drugs to produce local heat. Thermal energy is then used to selectively treat specific tumor tissue while avoiding overall temperature increases to reduce damage to the surrounding normal tissue. This is called magneto-chemotherapy (MCT) [93,94]. Phalake et al. [95] synthesized MIONPs by ferric chloride (III), manganese chloride (II), and ferric chloride (II) using the chemical precipitation method. These nanoparticles were then coated with chitosan to make CS-MIONPs. Doxorubicin (DOX), CS-MIONPs, and AS1411 aptamer were coupled to prepare Apt@DOX@CS-MIONP nanocomplexes. The nanocomplexes exhibited irregular spherical shapes and the particle size was 13.91 nm. The DOX release rate of the CS-MIONPs reached about 68.13% in a solution with a pH of 2.5. With increases in the pH, the release rate gradually decreased. DOX@CS-MIONPs exhibited significant cell killing, with cell killing rates of 71.48% and 92.2% in 2D and 3D MCF7 model culture after 24 h. Optical microscopy images demonstrated the tumor-targeting ability of the nanocomplexes. Without AMFs, Apt@DOX@CS-MIONP showed better biocompatibility. Under MCT for 30 min in tumor models, the apoptosis rate of cells was significantly increased and the tumor volume of the 3D MCF7 model was decreased.

#### Combination with sonodynamic therapy

Sonodynamic therapy (SDT) is a minimally invasive cancer treatment that involves the application of ultrasound and sonosensitizers to generate ROS such as singlet oxygen and hydroxyl radicals. ROS selectively target and impair cancer cells without harming normal tissues. The integration of high-intensity focused ultrasound (HIFU) into SDT targets energy more precisely and deeply, enhancing ROS production and overcoming the constraints of standard ultrasound in terms of depth. The use of Mn-based nanomaterials, such as MnO_2_, improves SDT by functioning as peroxidase mimics. These nanoparticles decompose hydrogen peroxide into oxygen, thus lowering hypoxia and preventing the restriction of ROS generation. These nanomaterials also serve as MRI contrast agents for real-time therapy monitoring and enable the controlled release of sonosensitizers, improving treatment accuracy and reducing side effects. Combining Mn-based nanomaterials with HIFU creates a multifunctional platform that not only penetrates deeper but also optimizes oxygen and ROS levels and provides imaging support. This offers a safer and more effective cancer treatment. The unique properties of these materials, including their responsiveness to the tumor environment, help overcome the limitations of traditional sonosensitizers, promising meaningful advances in SDT and better clinical outcomes.

Xu et al. [96] developed a core–shell structured nanoparticle loaded with IR780 and MnO_2_ (IR780/PLGA@MnO_2_). The authors performed in vitro experiments using the 4T1 mouse breast cancer cell line and observed the generation of singlet oxygen (^1^O_2_) as a measure of SDT efficacy. Singlet oxygen (^1^O_2_) possesses distinctive chemical properties and exhibits significant cytotoxicity to tumor cells, making it a ROS of importance. Upon ultrasonic irradiation at an intensity of 1 W/cm^2^ for 180 s, the relative efficiency of ^1^O_2_ production, calculated by the fluorescence intensity ratio F1 (under ultrasound irradiation) to F0 (before ultrasound irradiation), demonstrated a significant enhancement in the presence of IR780/PLGA@MnO_2_ (100 μg/ml) nanoparticles. Specifically, the fluorescence intensity of singlet oxygen sensor green increased to 819 at 180 s in the IR780/PLGA@MnO_2_ nanoparticle group without H_2_O_2_, whereas it rose remarkably to 1,150 in the presence of H_2_O_2_. The in vivo antitumor effects were evaluated in a 4T1 tumor-bearing mouse model, where the group treated with IR780/PLGA@MnO_2_ nanoparticles combined with ultrasound irradiation showed tumor growth inhibition, with the average tumor volume increasing only 5.5-fold after 14 days of treatment, compared to the saline group that exhibited a 7-fold increase (Fig. S3A).

Zhao et al. [97] developed a piezoelectric nanoplatform, M-BOC@SP, and assessed its SDT efficacy using 4T1 murine breast cancer cells (Fig. S3B). The platform obviously enhanced the generation of superoxide anions (O_2_^−^) and hydroxyl radicals (·OH) under ultrasound irradiation, as indicated by the absorbance of nitroblue tetrazolium (NBT) at 259 nm and the fluorescence intensity of terephthalic acid solution. In vivo, in a murine 4T1 breast cancer model, the M-BOC@SP+ultrasound group demonstrated a 70% tumor suppression rate, contrasting with the rapid tumor growth observed in the PBS group. The average tumor weight in the M-BOC@SP+ultrasound group was notably lower than that of the PBS group, highlighting the nanoplatform’s potent antitumor activity. Furthermore, the biocompatibility of M-BOC@SP was evaluated in healthy female Institute of Cancer Research (ICR) mice, with no adverse effects over a 14-day observation period, normal blood biochemistry and routine indices, and no histological damage in the major organs. These findings confirm the safety of this platform for potential clinical applications.

Zhang et al. [98] conducted in vitro studies to demonstrate the efficacy of RB@COF-MnO_x_-PEG (RCMP) for SDT on human osteosarcoma cells (MG-63) (Fig. S3C). Upon treatment with GSH and exposure to ultrasound, the RCMP group exhibited a significant decrease in the characteristic absorption of 1,3-diphenylisobenzofuran (DPBF) at 410 nm, indicating enhanced sonodynamic efficiency. Specifically, the absorbance of DPBF in the RCMP+ultrasound group decreased markedly after 5 min of ultrasound irradiation (1.0 W/cm^2^, 1.0 MHz, 50% duty cycle). Additionally, the RCMP+ultrasound group showed considerably higher fluorescence from the oxidized 2’,7’-dichlorodihydrofluorescein diacetate (DCFH) probe compared to the control and ultrasound-only groups. This suggests elevated levels of ROS. The RCMP+ultrasound group also displayed the weakest fluorescence in the Thiol Tracker Violet assay, indicating that this group exhibited the most significant depletion of intracellular GSH, with a fluorescence intensity significantly lower than the other groups. These findings highlight the potential of MnO_x_-coated COF nanobowls as activatable nanosensitizers for tumor-specific SDT in osteosarcoma treatment.

#### Combination with chemodynamic therapy

Chemodynamic treatment (CDT) has gained considerable interest in the last decade due to its high potential in tumor therapy. This is attributed to its ability to produce cytotoxic ROS directly at the tumor site while causing minimal harm to normal tissues. CDT harnesses the properties of the TME, including mild acidity and the raised H_2_O_2_ concentration, to trigger intracellular oxidative stress. To date, metal-based nanoparticles, including Mn^2+^, have been used as agents for cancer cell death therapy to induce ferroptosis through the use of natural H_2_O_2_ found in the body. Regrettably, the overexpression of GSH in tumor cells is a substantial challenge for CDT. GSH greatly enhances the resistance of tumor cells to oxidative stress and reduces the effectiveness of CDT [99,100]. Mn can regulate the TME within it and exert synergistic effects to enhance the antitumor capabilities of nanoparticles.

Gao et al. [101] synthesized chiral manganese dioxide (L/D-MnO_2_) nanoparticles by utilizing threonine molecules as chiral ligands. Due to the substantial specific surface area of L/D-MnO_2_, cisplatin was loaded to make L/D-MnO_2_@Pt nanoparticles (Fig. S4A). The concentration of Mn^2+^ was found to be 10,700 mg/l for D-MnO_2_ nanoparticles and 9,720 mg/l for L-MnO_2_ nanoparticles at a pH of 5.5. The L/D-MnO_2_@Pt nanoparticles were selectively taken up by cancer cells and effectively reduced the level of GSH through a redox process, resulting in the release of Mn^2+^ and Pt. The L/D-MnO_2_ nanoparticles and L/D-MnO_2_@Pt nanoparticles induced significant inhibition effects on 4T1 cancer cells, while there were nearly no effects on normal CHO cells. The Mn^2+^ that was liberated demonstrated a potent chemodynamic effect by means of a Fenton-like reaction. In addition, the reduction of GSH enhanced the effectiveness of CDT. An Annexin V–FITC/PI apoptosis detection kit was used to determine the percentages of apoptotic and necrotic cells treated with LMnO_2_@Pt nanoparticles and D-MnO_2_@Pt nanoparticles, which were 91.21% and 92.06%, respectively. These values were higher than the other groups. Thus, the integration of Mn^2+^ CDT with Pt chemotherapy notably improved the efficacy of tumor therapy.

Mn-based nanomaterials can not only catalyze the Fenton reaction and enhance the therapeutic effect of CDT but also induce the ICD of tumor cells. A nanozyme composed of a solitary Mn atom attached to a nitrogen-doped carbon (N/C) structure was created by Qiao et al. [102] (Fig. S4B). The TEM images showed that the size of the Mn-N/C nanozymes ranged from 600 to 700 nm, and they exhibited a uniform dodecahedral structure. Flow cytometry analysis showed a considerable increase in the ROS levels of MC38 colon cancer cells after treatment with Mn-N/C and H_2_O_2_. The combined application of Mn-N/C and H_2_O_2_ significantly reduced the viability of both CT26 and MC38 tumor cells over time. The mRNA levels of ICD markers such as Cxcl10, Hmgb1, and Ddit3 were considerably raised in tumor cells from Mn-N/C-treated tumors. Mn-N/C solution, diluted in saline (70 mg·kg^−1^, 50 μl), was administered intratumorally to the CT26 tumor-bearing C57BL/6J mice every 3 days. Mn-N/C therapy markedly inhibited CT26 tumor growth, diminished tumor weight, and extended survival in mice relative to the untreated and saline-treated groups. In terms of the mechanism, Mn-N/C therapy stimulated the activation of type I IFN signaling, which played a crucial role in the antitumor immune response induced by Mn-N/C.

In another study, Mn zinc sulfide nanomicelles were reprogrammed to inhibit the TME and thus block tumor cell metastasis in addition to inducing ICD and enhancing CDT. Li et al. [103] utilized an amphiphilic polyethylene glycol-poly(2-hexoxy-2-oxo-1,3,2-dioxaphospholane) copolymer with a thermally sensitive flowable core (mPEG-b-PHEP) to incorporate IR780 dye and Mn-doped zinc sulfide nanoparticles (ZMS) in order to form polymer micelles, referred to as PPIR780-ZMS (Fig. S4C). This allowed for the precise control of ZMS release upon NIR activation. The Mn release rate reached nearly 80% under NIR after 30 h. The production of ROS was enhanced by photothermal-triggered Mn^2+^-mediated CDT. The IC_50_ of the PPIR780-ZMS-treated group with NIR was decreased to 1.2 ± 0.3 μg/ml. These findings indicate that PPIR780-ZMS can enhance ICD in cancer cells, resulting in a high level of exposure to damage-associated molecular patterns.

#### Combination with immunotherapy

Cancer immunotherapy has emerged as a potent strategy for enhancing tumor treatment. Effective cancer immunotherapy aims to enable the patient’s immune system to eliminate tumor cells by intervening at different stages in the cancer-immunity cycle [104,105]. However, current immunotherapies are profoundly limited by their low response rate, which could be attributed to the immunosuppressive tumor microenvironment and the low infiltration of tumor-specific cytotoxic T lymphocytes (CTLs) such as regulatory T cells and tumor-associated macrophages [106,107]. The potential of metal-based agents for tumor immunomodulation (metalloimmunotherapy) has been recognized, and these metal ions, including Mn, have been shown to induce ICD in multiple tumor cells [108,109]. In particular, Mn-based agents can regulate the TME and create an immunoactive milieu through the cGAS-STING-TLR4-related signaling pathways, among others. Among these, the effects of Mn-based agents on the cGAS-STING pathway have been investigated most extensively. Therefore, here we focus on the application of Mn-based nanomaterials to regulate the cGAS-STING pathway in immunotherapy.

Cheng et al. [110] synthesized multifunctional hybrid exosomes for cGAS/STING activation (SN/Mn@gHE). Specifically, the authors fused exosomes carrying CD47, which had great tumor-targeting capacity, and exosomes from M1 macrophages. These were finally coated with DNA-targeting agent (SN38) and a STING agonist (MnO_2_) (Fig. S5A). The morphology, size, pH-responsiveness, phagocytic activity, and cellular internalization of the SN/Mn@gHE nanoparticles were evaluated. The results showed that they had a better MnO_2_ release capacity at a pH of 6.5 and gHE was able to target 4T1 cells. gHE increased the rate of CD80^−^- and CD86^−^-positive cells to 88.9%, indicating that gHE induced M2 macrophage repolarization in vitro. The cell apoptosis rate of 4T1 cells in the SN/Mn@gHE (pH 6.5) group was 26.73%, as compared with the free SN38 group of 12.64%. Furthermore, SN/Mn@gHE promoted the maturation of DCs and facilitated CTL infiltration and NK cell recruitment to the tumor, leading to significant antitumor and antimetastatic efficacy. In mice bearing 4T1 tumors, SN/Mn@gHE killed tumor cells and decreased the tumor volume by eliciting immune responses related to cGAS/STING activation.

The combination of metal immunotherapy and chemotherapy has also attracted the attention of researchers. Luo et al. [111] developed a highly advanced nanoplatform to specifically respond to the TME. The nanoplatform was shown to alleviate hypoxia and stimulate the immune system. The MnO_2_@OxA@OMV nanoreactor contained MnO_2_ nanoenzymes wrapped in bacterial-derived outer membrane vesicles (OMVs) and the ICD inducer oxaliplatin (OxA) (Fig. S5B). This nanoreactor exhibited inherent catalase-like activity within the TME, efficiently converting endogenous H_2_O_2_ into O_2_. After 24 h, its release rate reached as high as about 98.5%. This process enabled a sustained oxygen supply, reducing the tumor’s oxidative stress and hypoxic TME, and accelerating the release of OxA. The synergistic effect of OxA-induced ICD and Mn^2+^ release from the nanoreactor enhanced the stimulation of the cGAS-STING pathway, leading to a substantial increase in the activation of STING and the maturation of DCs. The MnO_2_@OxA@OMV group exhibited the highest DC maturation rate of about 39.60%, which was twice that of the control group. The in vitro antitumor efficacy of MnO_2_@OxA@OMV to CT26 cells reached about 50% after 24 h. This ultimately resulted in the effectiveness of the metalloimmunotherapy. In addition, the immunostimulant OMVs played a vital role in facilitating the infiltration of activated CD8^+^ T cells into the solid tumor. The ratio of CD8^+^ T cells was markedly elevated in the MnO_2_@OxA@OMV group, with a 2.6-fold rise in CD8^+^ T cells relative to the control group. Similar results were observed in CT26 colorectal tumor-bearing mice. Similarly, Wang et al. [112] created hydrogenated manganese oxide nanoparticles (mHMnO-DOX) that were loaded with DOX and modified with the membrane of 4T1 cancer cells. The mHMnO-DOX treatment effectively decreased the initial and distant tumor growth, prevented tumor relapse and metastasis, and significantly increased the longevity of 4T1 tumor-bearing mice.

Among the available studies of the regulation of the cGAS-STING pathway by Mn-based nanoparticles, the participation of other metals has been shown to exert a synergistic effect on the nanoparticle’s antitumor ability. Du et al. [113] prepared a nanovaccine consisting of Mn-doped mesoporous silica as a carrier (MSN). It was loaded with sorafenib (SOR) and modified with MIL-100 (Fe) and was named MF@SOR (Fig. S5C). Upon applying a layer of MIL-100 (Fe) onto the MSN, the pores of the MSN were completely sealed, resulting in a significant increase in the size of the nanoparticle, to around 153.87±1.8 nm. The release of Fe^3+^, SOR, and Mn^2+^ into the tumor was coordinated and facilitated by the TME. About 65% of the SOR was discharged from the MF@SOR nanovaccine following a 48-h incubation period in PBS at a pH of 5.0 with 10 mM GSH. Fe^3+^ effectively worked with SOR-induced immunogenic pyroptosis through both the classical and nonclassical signaling pathways, leading to the release of a large amount of immunogenic factors. Hepa1 to 6 cells were labeled with an anti-creatine transporter antibody, and distinct green fluorescence signals were observed in the MF@SOR-treated group, with less intense green fluorescence signals observed in the other groups, including the MSN@SOR-treated group. This process promoted the maturation of DCs and the exposure of dsDNA, which was demonstrated in CD11c^−^-, CD80^−^-, and CD86^−^-positive cells. The proportions of CD80^+^, CD86^+^, and CD11c^+^ DCs in the MF@SOR (69.07 ± 1.11%) group exhibited a sequential increase compared to the control group (43.63% ± 0.39%) and MSN@SOR group (62.77% ± 0.63%). This was attributed to the pyroptosis-mediated release of proinflammatory substances and the activation of the cGAS-STING pathway.

#### Combination with gene therapy

Tumor gene therapy has attracted much attention in recent years due to its novel and effective properties. By changing gene expression, this approach can suppress tumor growth, promote tumor cell death, or enhance the body’s immune response to a tumor. Gene therapy strategies mainly include gene replacement, gene repair, gene modification, gene inactivation, immune regulation, etc. The key to gene therapy lies in the use of an effective carrier system to deliver therapeutic genes into tumor cells. Nanoparticles, as nonviral carriers, have good biological compatibility and low immunogenicity. The pressing issue in gene therapy with the use of Mn-based nanomaterials is to harness their synergistic effect with target genes.

The adhesion molecule CD44, a cell surface type 1 hyaluronic acid transmembrane glycoprotein receptor, is highly expressed in ovarian cancer, and its aberrant expression plays a crucial role in the occurrence, development, invasion, and metastasis of ovarian cancer [114]. Guo et al. [115] prepared PEG-modified Mn zinc ferrite nanoparticles (PEG-MZF-NPs) to support cisplatin (DDP) and CD44-shRNA nanoliposomes, and these were bound together by the thin film-ultrasonic method and high-speed stirring. The resulting nanoliposomes were called PEG-MZF-NPs/DDP/CD44-shRNA (Fig. S6). The nanoliposomes were 15 to 25 nm in diameter according to TEM. Under the action of an AMF, the proliferation inhibition rate of ovarian cancer HO8910 cells treated with PEG-MZF-NPs/DDP/CD44-shRNA was 91.33% ± 0.22%, which was significantly higher than the other groups. Moreover, the nanoliposomes were able to restrain the proliferation and invasion of tumor cells and promote cell apoptosis. In addition to downregulating the expression of the CD44, VEGF, survivin, BCL-2, and BCL-xl proteins, the caspase-3 and caspase-9 proteins were significantly increased both in vitro and in vivo. These effects were far superior to other individual therapies. Moreover, the combination therapy had no significant effect on bone marrow hematopoiesis and liver and kidney function in nude mice, demonstrating its biological safety.

Pyroptosis is a proinflammatory form of programmed cell death that has been widely reported in cancer. The pyroptosis-executed protein GSDME is suppressed in many cancers including triple-negative breast cancer (TNBC) [116]. To combine gene and immune therapy, Zhong et al. [117] constructed a cationic nanoliposome based on 2,3-dioleoyloxy-propyl-trimethylammonium-chloride and DOPE (GM@LR) to codeliver the GSDME-expressing plasmid and manganese carbonyl (MnCO) into TNBC cells. The structure, loading capacity of MnCO, hydrodynamic diameter, and stability of nanodrugs were characterized by UV–Vis spectrophotometry, agarose gel electrophoresis, DLS, and zeta potential analysis. The in vitro release of MnCO from GM@LR was 43.4% of the MnCO released within 24 h. GM@LR with oxazole yellow homodimer (YOYO-1)-labeled pGSDME was internalized by 4T1 cells. While G@LR did not induce any obvious cell death, MnCO-loaded nanodrug treatments (M@LR and GM@LR) decreased the viability of 4T1 cells to about 60%. The mechanisms involved the induction of cell pyroptosis and the activation of the STING pathway. In orthotopic breast tumor-bearing mice, the antitumor efficacy of GM@LR was significantly increased and the survival of tumor-bearing mice was prolonged. This was attributed to immune activation due to Mn^2+^-dependent activation of the cGAS-STING signaling pathway. Further, MnCO endowed the sensitivity of GM@LR-dependent MRI in an intrahepatic metastatic tumor model, and the antimetastatic effect of the nanodrugs was increased.

#### Combination with intervention therapy

Tumor intervention therapy is a new type of treatment that uses minimally invasive methods to directly treat tumor lesions through small blood vessels or surface channels. This approach is guided by imaging equipment such as x-rays, CT, MRI, etc. The advantage of this method lies in its minimally invasive nature, high safety, fast patient recovery, and the ability to treat tumors in a targeted manner. It is especially suitable for patients with tumors that are difficult and high risk to treat surgically. According to the different mechanisms of tumor killing, this approach can be mainly divided into arterial infusion chemotherapy, vascular embolization therapy, tumor ablation, implant therapy, and interventional gene therapy. Microwave ablation (MWA), one such intervention therapy, is a widely utilized minimally invasive therapeutic technique for numerous solid tumors throughout the entire body, including liver cancer, renal carcinoma, thyroid papillary carcinoma, and breast cancer [118,119]. Specifically, this technique employs the tissue heating effect produced by microwave energy (300 MHz to 300 GHz) to address solid tumors, making it especially appropriate for patients who are ineligible for surgical intervention.

Zhou et al. [120] offered a hopeful approach for completely eliminating any remaining tumor after MWA by integrating MWA with CDT. The authors reported a simple and efficient Mn hydrogel (ALG-Mn hydrogel) that was synthesized using a straightforward mixing technique. The hydrogel demonstrated exceptional syringeability, notable microwave responsiveness, and powerful Fenton-like reactivity. The CT26 cell viability significantly decreased to 20% after an additional 24-h incubation of the ALG-Mn hydrogel with high-power MWA (4 W, 5 min), compared with the 50% cell viability in the MWA-only group. The synergistic antitumor effect of the ALG-Mn hydrogel in the CT26 tumor-bearing mouse model was similar. Importantly, the Mn in the ALG-Mn hydrogel could be monitored by MRI in vivo. The kidneys in the ALG-Mn hydrogel-treated animals showed an increase in signal intensity 1 h after injection, with the highest point reached after 6 h. It then progressively decreased over a period of 2 days, which was longer than the MnCl_2_-treated groups.

### Application of Mn-based nanomaterials in tumor diagnosis

At present, the techniques used for diagnosing tumors include blood tests, imaging tests, pathology tests, and endoscopy. Among these, imaging tests have emerged as a crucial approach for diagnosis since they allow for visual evaluation of the tumor’s location, size, and influence on surrounding tissues. In particular, MRI is extensively utilized for diagnosing several types of tumors, including neurological, head and neck, breast, abdominal, and pelvic tumors. Conventionally, clinical MRI studies frequently employ gadolinium (Gd) as a contrast agent. However, the utilization of Gd contrast agents is restricted due to their potential to cause kidney damage. As an alternative, Mn^2+^ has been developed as a T1 MRI contrast agent due to its paramagnetic properties. Although Mn^2+^ avoids nephrotoxicity, its potential to cause acute cardiac injury limits its clinical use [121]. The development of Mn-based nanomaterials offers a solution. These nanoparticles mitigate the risk of acute cardiac injury by reducing the biotoxicity of Mn^2+^ and extending its circulation time in the body. In addition, Mn nanoparticles can be used as drug carriers, which allows them to be used not only for the diagnosis of tumors but also for their treatment. This allows for the integration of tumor diagnosis and treatment. By functionalizing these nanoparticles, targeting and monitoring of tumors can be achieved, further enhancing the accuracy of diagnosis. At the same time, the use of Mn nanoparticles may also support multimodal imaging techniques, when combined with techniques such as PET or CT, to provide more comprehensive tumor diagnostic information for doctors. Mn-based nanomaterials demonstrate great potential in tumor diagnosis and treatment, especially in improving MRI diagnostic accuracy and reducing potential toxicity. Therefore, the next section will focus on the application of Mn-based nanomaterials for enhancing contrast and multimodal imaging.

#### MRI contrast enhancement with Mn-based nanoparticles

**Fig. 4.**
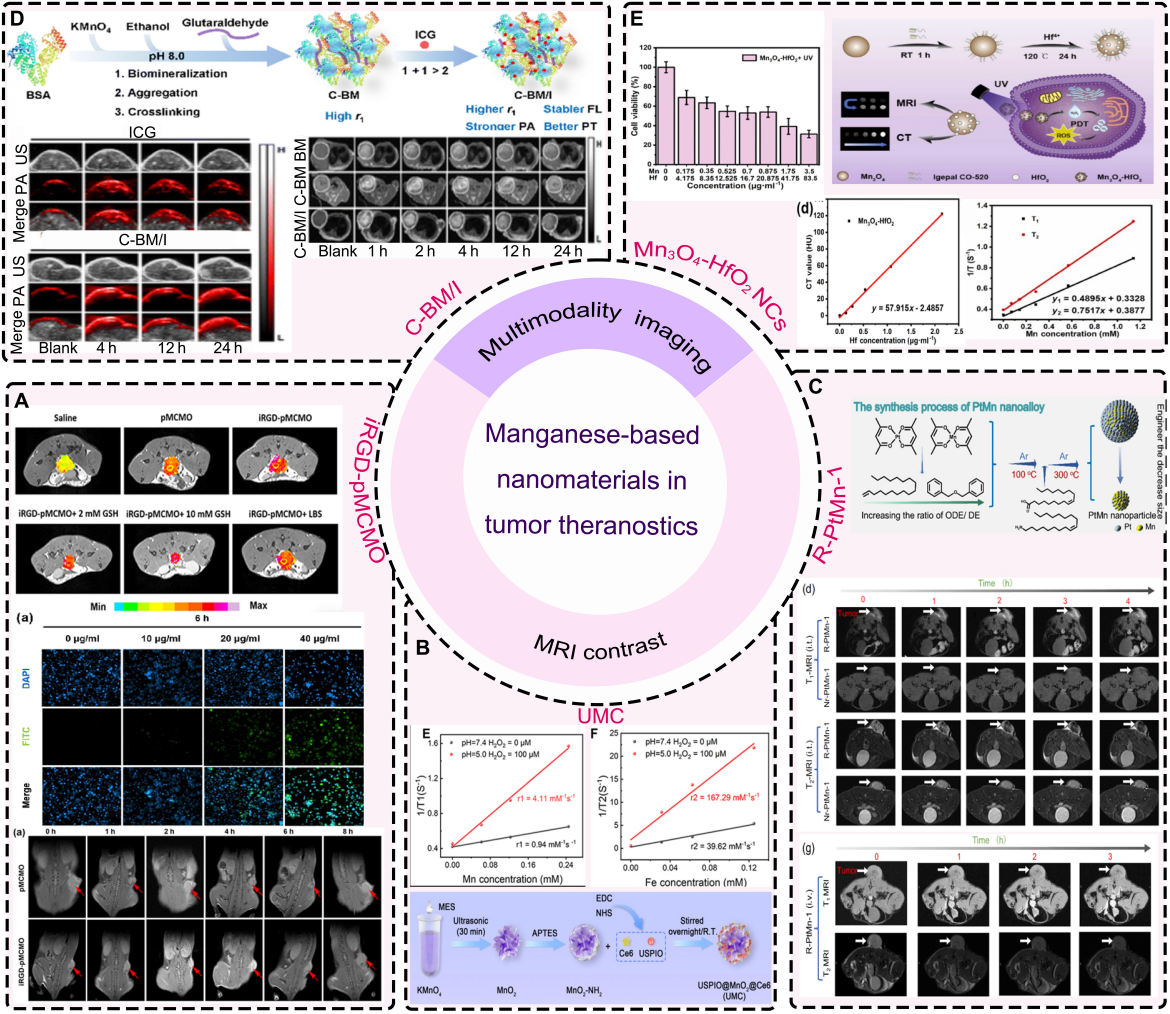
Mn-based nanomaterials in tumor theranostics. (A) iRGD-pMCMO; (B) UMC; (C) R-PtMn-1; (D) C-BM/I; (E) Mn_3_O_4_-HfO_2_ NCs. Panel (A) is adapted with permission from Ref. [122], copyright 2024 *International Journal of Nanomedicine*. Panel (B) is adapted with permission from Ref. [123], copyright 2022 *International Journal of Nanomedicine*. Panel (C) is adapted with permission from Ref. [124], copyright 2023 *Journal of Nanobiotechnology*. Panel (D) is adapted with permission from Ref. [126], copyright 2023 *Journal of Materials Chemistry*. Panel (E) is adapted with permission from Ref. [127], copyright 2024 *Colloids and Surfaces B: Biointerfaces*.

Yang et al. [122] developed an αv integrin receptor-targeting multi-crystalline manganese oxide (iRGD-pMCMO) as a novel MRI contrast agent and evaluated the MRI contrast effects of iRGD-pMCMO nanoparticles in the PC-3 tumor cell line (Fig. 4A). These nanoparticles were designed to enhance tumor-selective imaging by targeting αv integrin receptors. The study found that the T1 relaxation rate of iRGD-pMCMO was 1.28 mM^−1^ s^−1^ under neutral conditions, but significantly increased to 2.5 and 5.69 mM^−1^ s^−1^ at a pH of 6.5 and 5.5, respectively. Further experiments showed that upon the addition of GSH, the T1 relaxation rate increased to 8.54 mM^−1^ s^−1^ at a pH of 5.5, and sharply rose to 17.60 mM^−1^ s^−1^ at a GSH concentration of 10 mM. These results indicate that iRGD-pMCMO serves as an excellent tumor-selective imaging contrast, with the potential for accurate MRI diagnosis of tumors.

Lv et al. [123] fabricated a honeycomb-like MnO_2_ to coload chlorin e6 and ultrasmall superparamagnetic iron oxide (USPIO@MnO_2_@Ce6, UMC) as a multifunctional nano-snowflake probe for enhanced PDT and multimodal imaging-guided tumor therapy (Fig. 4B). The authors discovered that the r1 relaxation rate of UMC increased from 0.94 to 4.11 mM^−1^ s^−1^ in the presence of H_2_O_2_, indicating a significant enhancement in T1-weighted imaging. Similarly, the r2 relaxation rate increased from 39.62 to 167.29 mM^−1^ s^−1^, demonstrating a substantial effect on T2-weighted imaging. These changes in the relaxation rates highlight the UMC probe’s ability to enhance MRI contrast, thereby improving the visibility of tumors and the precision of targeted treatments.

Guan et al. [124] investigated the MRI enhancement effects of R-PtMn-1 (PtMn nanoparticles with an acidity-responsive polymer) in an acidic TME. This nanoalloy was chosen for its ability to respond to the acidic conditions commonly found in tumor tissues (Fig. 4C). The experimental results showed that R-PtMn-1 significantly enhanced both T1 and T2 MRI contrast at a pH of 6.8, with T1 and T2 values increasing 3-fold and 3.2-fold, respectively. Under acidic conditions (pH 5.4), R-PtMn-1 rapidly released a large amount of Mn ions, further improving the MRI contrast. In vivo experiments with 4T1 tumor model mice demonstrated that intravenous injection of R-PtMn-1 enhanced T1-MRI signals and decreased T2-MRI signals, highlighting its effectiveness in tumor diagnosis. The use of both T1 and T2 values provides a comprehensive assessment of the contrast agent’s performance in different relaxation environments, reinforcing the potential of R-PtMn-1 for clinical MRI applications.

Gowtham et al.[125] developed carbon-coated Mn ferrite nanoparticles (MNF@Cs), which were further enhanced with an alginate derivative to create MNF@C-OSAs, a multimodal contrast agent for medical imaging. These novel particles showed improved superparamagnetism and fluorescence, offering a less toxic and more cost-effective alternative to traditional Gd-based agents for MRI. In vitro, MNF@C-OSAs demonstrated greater multimodal efficacy than MNF@C particles and conventional agents. Their relaxivities were 8.9 mM^−1^ s^−1^ for MNF@Cs and 8.2 mM^−1^ s^−1^ for MNF@C-OSAs, suggesting their suitability as T1 MRI contrast agents. T2-weighted images revealed negative contrasts, with r2 values of 44.54 mM^−1^ s^−1^ for MNF@Cs and 42.19 mM^−1^ s^−1^ for MNF@C-OSAs, and relaxivity ratios of 5.01 and 5.14, respectively. This highlights their potential for dual-mode T1 and T2 imaging. MNF@C-OSAs also demonstrated good biocompatibility and low toxicity in A549 cells and zebrafish embryos, supporting their use as theranostic agents.

These studies suggest that Mn-based nanomaterials have substantial potential for enhancing MRI contrast and improving tumor diagnosis, with promise for future clinical applications.

#### Multimodal MRI with Mn-based nanoparticles

An et al. [126] formed the C-BSA-MnO_2_/ICG nanocomposite (C-BM/I) by loading indocyanine green (ICG) into cross-linked BM nano-aggregates and performed experiments in H460 lung cancer cells and H460 tumor-bearing model mice (Fig. 4D). The authors found that the C-BM/I nanoprobe showed high MR signal enhancement (SE), relatively stable near-infrared fluorescence (NIR FL) intensity upon 808-nm laser irradiation, a sensitive photoacoustic (PA) response, and strong photothermal–photodynamic injury ability to the cancer cells. The T1 relaxivity of C-BM was 67.2 mM^−1^ s^−1^, and after loading ICG, the T1 relaxivity of C-BM/I reached 97.3 mM^−1^ s^−1^. These results indicate that the C-BM/I nanocomposite could be used for sensitive T1-weighted MR, NIR-II FL, and PA multimodal imaging and highly efficient cancer phototherapy.

Cui et al. [127] developed Mn-hafnium nanocomposites (Mn_3_O_4_-HfO_2_ NCs) for dual-modal MRI/CT imaging and PDT of colon cancer (Fig. 4E). These nanocomposites enhanced T1-weighted MRI and x-ray CT images, proving effective as molecular imaging probes with good relaxivities. The T1 MRI intensity increased with higher Mn levels, yielding relaxation rates of 0.4985 and 0.7517 mM^−1^ s^−1^ for r1 and r2, respectively. In vitro tests on the NCTC clone 929 (L929) and CT26 mouse colon cancer cells showed that the nanocomposites were nontoxic and biocompatible. When activated by low-intensity UV light (6 mW cm^−2^), the Mn_3_O_4_-HfO_2_ nanocomposite efficiently generated ROS, effectively targeting colon cancer cells. These findings highlight the potential of these composites in cancer diagnostics and PDT.

## Conclusion and Perspectives

In this review, we have extensively discussed the last decade’s advances in the development of Mn-based nanomaterials for the treatment and diagnosis of tumors. Due to the complexity of tumor treatment and the obscurity of diagnosis, one kind of therapy alone cannot completely eradicate all malignancies. Combination therapy can compensate for the defects of monotherapy and improve the efficiency of treatment [128,129]. The application of Mn-based nanomaterials in cancer treatment has mainly been combined with existing cancer treatment methods and new treatment methods, such as chemotherapy, RT, gene therapy, and interventional therapy (specific examples are summarized in Table). The advantages of Mn-based nanomaterials in cancer treatment include 3 aspects [18,145,146]. Mn-based nanomaterials, as carriers of tumor therapies, can carry chemotherapy drugs, photosensitizers, gene therapy molecules, etc. They can reduce the removal of drug molecules by the blood and improve the targeting of tumor tissue and the uptake and release of drugs through surface modification. Mn-based nanomaterials can be used as immune activators to activate innate immunity through immune-related signaling pathways such as cGAS-STING. They play a role in synergistic immunotherapy and improve the antitumor effect of the treatment. Mn-based nanomaterials act as regulators of the TME by consuming tumor local GSH and H_2_O_2_, increasing ROS, and inhibiting tumor proliferation through apoptosis, ferroptosis, or other mechanisms. Furthermore, Mn-based nanoparticles are advantageous in tumor diagnosis as it has magnetic properties that enhance the imaging contrast of MRI and can be harnessed to achieve multimodal imaging with fluorescence, ultrasound, and CT.

**Table.** Summary of application of manganese-based nanomaterials in tumor therapy

Name of nanomaterials	Size (nm)	Cell type	Cell safety concentration in vitro (incubation time)	Animal tumor model	Cell safety concentration in vivo (incubation time)	Performance	Application(s)	Ref.
MnO_2_PDA@Lipo@Geb@Beb	5 nm	A549	50 μg/ml (24 h)	A549 tumor-bearing mice	Geb = 3 mg/kg (20 days)	Tumor inhibition rate in A549 tumor-bearing mice of 78.54%	CHEMO	[68]
Mn-D@BPFe-A NPs	20–40 nm	HepG2	1 μg/ml (24 h)	(ICR)HepG2 tumor-bearing mice	40 mg/kg (CMn, 3.44 mg/kg, CDOX, 2.47 mg/kg, 15 days)	Tumor mass decreased from 2.5 to 1 g	CHEMO	[69]
CD147-MTRN/DOX	Unknown	Huh7	1,000 μg/ml (48 h)	Huh7 tumor-bearing nude mice	MTRN an amount of 19,000 μg/kg (18 days)	Tumor volume decreasedby 85%	CHEMO	[71]
		L02						
MnO_2_–Pt(IV) NPs	135±24 nm	A549	100 μM (48 h)	A549 tumor-bearing nude mice	6 mg/kg (24 h)	T1 signal enhanced to 240%	CHEMO/MRI	[70]
CuS@mSiO_2_@MnO_2_	30 nm	HeLa	100 μg/ml (24 h)	HeLa tumor-bearing mice	20 mg/kg (14days)	The apoptosis rate of tumor cells was 18.3%	RT	[130]
αPDL1@MnO_2_	100 nm	CT26	50 μM (24 h)	CT26 tumor-bearing mice	10 mg/kg (7 days)	The maturation rate of DC cells was 42.2%	RT	[131]
Bio-MnO_2_ NPs	101 nm	A549	25 μg/ml (24 h)	LLC tumor-bearing mice	2 mg/kg (7days)	Ratio of CD45^+^ CD3^+^ CD8^+^ and CD45^+^ CD3^+^ was 65.6%	RT	[73]
		PC9						
		H520						
		LLC						
HGMP NPs	69.07 ± 4.10 nm	4T1	4.5 μg/ml (24 h)	4T1 tumor-bearing Balb/c mice	2 mg/kg (14 days)	The survival rate of tumor cells decreased to 30.3%	RT	[76]
PEG-MnO_2_-Ce6 (PMC) NPs	73.69 ± 1.20nm	B16-F10	PMC (Ce6: 10 μg/ml, Mn^2+^: 15 μg/ml, 24 h)	B16F10 tumor-bearing mice	Unknown	The proportion of CD8^+^ T cells in primary tumor tissue increased as 31.71% ± 2.11%	PTT (660nm)	[132]
rGO@MnO^2^/MB/Dox	150 nm	PC-12	150 μg/ml (12 h)	CBT tissue tumor-bearing nude mice	100 mg/ml (14 days)	The tumor volume was reduced by more than 50%	PDT/PTT (808 nm)/CHOME	[133]
UCSMn	65 nm	MCF-7	800 μg/ml (6 h)	MCF-7 tumor-bearing nude mice	Unknown	The average tumor volume dropped from 500 to 100 mm^3^	PDT (450/980 nm)	[134]
mPDA/Cur@M/CM	389.6 ± 19.5 nm	4T1	50 μg/ml (24 h)	CAFs and 4T1 tumor-bearing mice	Curcumin = 20 mg/kg, mPDA = 25 mg/kg (16days)	Tumor fluorescence signal increased 2.34 times	PTT (808 nm)/CHEMO	[135]
ICG@MnO_2_@Exo-anti-PD-L1 NPs	100 nm	LLC	2 μg/ml (24 h)	Lewis tumor-bearing C57 mice	3 mg/kg (16 days)	The tumor was significantly reduced or even eliminated	PDT (808 nm)	[136]
HRG-Mn_3_O_4_	12 ± 2.21 nm	A549	100 μg/ml (24 h)	Not test	Not test	The survival rate of tumor cells decreased from 100% to 30%	PDT (670 nm)	[83]
MnO_2_ nanoflower	45 nm	HeLa	50 μg/ml (24 h)	Not test	Not test	The tumor cells are completely killed	PTT/PDT (808 nm)	[77]
rGOQD/IR820/MnO_2_/CPP	80–100 nm	3T3	100 μg/ml (24 h)	U87 cell orthotopic brain tumor xenograft model	0.5 mg/ml (14 days)	The tumor temperature increased from 34 °C to 51 °C after 5 min	PTT/PDT (808 nm)	[80]
		U87						
MoO_3-x_-Ag-PEG-MnO_2_	50 nm	HeLa	200 μg/ml (24 h)	HeLa tumor-bearing nude mice	5 mg/kg (14 days)	The tumor tissue was completely ablated	PTT/PDT (808 nm)	[86]
		MCF-7						
Brusatol/silica@MnO_2_/Ce6@PDA (BSMCP)	100 nm	Mia-PaCa-2	400 μg/ml (6 h)	Mia-PaCa-2 tumor-bearing nude mice	100 μg/kg (12 days)	Tumor temperature increased from 31 °C to 52 °C in 10 min	PT (660 nm/808 nm)	[137]
L/D-MnO_2_@Pt NPs	160 nm	CHO	128 μg/ml (24 h)	Not test	Not test	The necrosis and apoptosis rates of tumor cells were 91.21% and 92.06%, respectively	CDT	[101]
		4T1						
MOC-Mn	100 μm	HeLa	125 μg/ml (24 h)	HeLa tumor-bearing mice	0.015 mM, 100 μl (14 days)	The tumor volume in the mice increased only 1.9 times, compared with 14.2 times in the control group	CDT	[138]
		HK2						
Mn(II) complex	Unknown	MCF7	62.5 μg/m l(24 h)	TUBO tumor-bearing mice	12.5 mg/kg (14 days)	The cell cycle of 76.8% of tumor cells remained in G1 phase	EMT	[92]
		TUBO						
Cs-MIONPs	16.88 nm	MCF-7	1 mg/ml (24 h)	Not test	Not test	No clear statistics	EMT	[95]
IR780/PLGA@MnO_2_ NPs	300.34 ± 4.56 nm	4T1	6.25 μg/ml (24 h)	4T1 tumor-bearing mice	2.0 mg/ml, 200 μl (14 days)	The tumor volume increased more slowly than the control group	SDT (1.0 W/cm^2^)	[96]
		MCF-7						
RB@COF-MnO_x_-PEG	149 nm	MG63	60 μg/ml (24 h)	HOSMNNG tumor-bearing mice	10 mg/kg (14 days)	Tumor growth inhibition rate reached 74%	SDT (1.0 W/cm^2^)	[98]
M-BOC@SP NSs	Side length 150 ± 20 nm	L929	200 μg/ml (24 h)	4T1 tumor-bearing mice	200 mg/kg (14 days, ICR mice)	Tumor inhibition rate reached 70%	SDT (1.0 W/cm^2^)	[97]
	Thickness 15 ± 2 nm	4T1						
CPT nps	340 nm	A549	20 μg/ml (24 h)	LLC tumor-bearing BALB/c mice	2 mg/kg (14 days)	After 14 days, the volume of tumor was 200 mm^3^, compared to 1,000 mm^3^ in the control group	CPT	[139]
		LLC						
MnO_2_-Al-OVA	176.0 ± 1.9 nm	DC2.4	70 μg/ml (24 h, DC2.4)	C57 transplantation tumor model mice with EG7-OVA cells	MnO_2_-Al-OVA (50 μg MnO_2_, 1 μg Al, 10 μg OVA) 50 μl (35 days)	Tumor growth was almost completely suppressed	IMT	[140]
		L929	200 μg/ml (24 h, L929)					
Mn-N/C	600–700 nm	CT26	100 μg/ml (24 h)	CT26 tumor-bearing Balb/c mice	70 mg/kg (20 days)	The tumor volume was reduced to 500 mm^3^, compared to 1,500 mm^3^ in the control group	IMT/CDT	[102]
		MC38		MC38 tumor-bearing C57mice				
MPCZ NPs	30 nm	4T1	200 μg/ml (16 h)	4T1 tumor-bearing mice	20 mg/kg (14 days)	The percentage of CD8^+^ cells reached 22.9%, the percentage of CD4^+^ cells reached 47.4%, and the percentage of DC cells was 2.7%	IMT	[141]
M-G/CDz@B NPs	65 nm	B16F10	50 μg/ml	B16F10 tumor-bearing mice	100 μg/ml (24 h, RBC)	The ratio of CD3^+^CD8^+^CD4^+^CD62L^−^ cells reached 23.1%	IMT	[142]
		4T1						
MHN NGs	186.5 ± 10.4nm	3T3	40 μg/ml (24 h)	B16F10 tumor-bearing mice	10 mg/kg (9 days)	The amount of accumulation in the tumor is 8 times that of the control group, which has the strongest tumor inhibition effect	IMT	[143]
		B16F10						
Fe-MnO_2_/DHA	200 nm	Hep1–6	14.01 μg/ml (24 h HepG2)	Hepa1–6 tumor C57BL/6 mice	250 μg/ml (2 h, RBC)	The total apoptotic cell ratio of Hepa1–6 and HepG2 cells was 64.2% and 66.22%	IMT	[72]
		HepG2	10.17 μg/ml (24 h Hep1–6)					
M-M NPs	2 nm	B16, B16-f10, B16-OVA,MC38, MC38-OVA,MB49	200 μM (1 h)	Unilateral tumor model with B16, MC38, or of MB49 cells on the left flank; bilateral tumor model with B16, MC38-OVA, or MC38 cells on the right flank and B16, B16-OVA, or B16-OVA on the corresponding left flank	10 mg/kg (10 days)	Tumor inhibition rate was over 50%	IMT	[144]
PEG-MZF-NPs/DDP/CD44-shRNA nanoliposomes	15–25 nm	HO8910	Unknown	HO8910 tumor-bearing nude mice	10 ml/kg (6 weeks)	The tumor volume inhibition rate was (92.80 ± 1.09) %	GENE/MFH (235 kHz 35A)/CHEMO	[115]
GM@LR	108 nm	4T1	Unknown	4T1 tumor-bearing Balb/c mice orthotopic breast transplantation model	MnCO 1.5 mg/kg, pGSDME 20 μg/mouse (22 days)	The induction rate of early apoptotic cells was 31.6%, and the induction rate of late apoptotic cells was 17.6%	GENE/IMT	[117]
ALG-Mn hydrogel	20–50 mm	HepG2	10 mM (24 h)	CT26 tumor-bearing mice	ALG: 20mg/ml, Mn^2+^: 50 mm (14 days)	The tumor was almost completely suppressed	MWA	[120]
		CT26						
iRGD-pMCMO	260 nm	PC3	100 μg/ml (24 h)	Injection of 107 tumor cells into the right legs of mice. TRAMP model mice	50 mg/kg (SD rats)	Four hours after injection, the maximum MRI signal strength of the tumor increased to 229 units	MRI	[122]
		THLE-3						
		C166						
R-PtMn-1	2.7 ± 0.6 nm	4T1	100 μg/ml (24 h, 4T1)	CT26 or 4T1 tumor-bearing mice	200 μl, Mn: 0.7 mg/ml (14 days)	R-PtMn-1 has a good enhancement ratio (pH 6.4/pH 7.4), 3× or 3.2x for T1 or T2 MRI, respectively	MRI	[124]
		CT26						
		HEK293						
USPIO@MnO_2_@Ce6	142 nm	4T1	5 μg/ml (24 h)	4T1 tumor-bearing mice	20 mg/kg (MnO_2_), 20 mg/kg (USPIO), 16 mg/kg (Ce6) 14 days	Tumor cell inhibition rate was 70%	MRI/PDT (660nm)	[123]
MNF@C-OSA	140.7 nm	A549	20 mM (24 h)	Zebrafish embryos	20 mM (72 h)	Imaging capability was 2.877 × 10^8^ p/s/cm^2^/sr per mM	MRI	[125]
C-BM/I	3.2 nm	H460	10 μg/ml (24 h)	H460 tumor-bearing mice	80 μg/ml [ICG], 50 μl (2 h)	The maximum temperature during the 5-min exposure period was 51.5	MRI/PTT (808 nm)	[126]
Mn_3_O_4_-HfO_2_NCs	15 nm	CT26 L929	Mn: 3.5 μg/ml, Hf: 83.5 μg/ml (24 h)	Not test	Not test	ROS production (%) increased by more than 400%	MRI/PDT (6 mW/cm)	[127]

CHEMO, chemotherapy; RT, radiation therapy; PTT, photothermal therapy; PDT, photodynamic therapy; PT, phototherapy; EMT, electromagnetic therapy; SDT, sonodynamic therapy; CDT, chemodynamic therapy; CPT, chemo-photothermal therapy; IMT, immunotherapy; GENE, gene therapy; MFH, magnetic fluid hyperthermia; MWA, microwave ablation; MRI, magnetic resonance imaging.

Although the synthesis of Mn-based nanocomposites and applied research in tumor diagnosis and treatment have developed rapidly in recent years, no studies have been reported yet on clinical trials. Only one phase 1 clinical trial has been reported to evaluate the efficacy and safety of Mn^2+^ priming anti-PD-1 antibody plus chemotherapy [147]. Therefore, the clinical application of these materials has many challenges to overcome. The most important challenge is that the effect of Mn on tumors needs to be further clarified, and some studies have shown that Mn has a promoting effect in some tumors [148]. In particular, reports have shown that Mn^2 +^ exists in the TME, but a high dose of Mn^2 +^ has been shown to be cytotoxic. Mn^2+^ is mainly enriched in the brain, so its dosage and biological safety need to be further explored. Although many studies have shown that most Mn-based nanosystems have good biocompatibility and low toxicity, their long-term toxicity has not been fully evaluated. Its toxicity must be reduced and its biodegradation must be accelerated for clinical application [149,150]. In addition, in order to combine Mn with a variety of treatments, various functional components, such as inorganic particles, biological molecules, and polymer molecules, need to be added to the preparation process. The different components of Mn nanomaterials will bring new challenges to the stability and reliability of preparation. It is necessary for researchers to develop large-scale, high-quality production processes and reduce production costs.

**Fig. 5.**
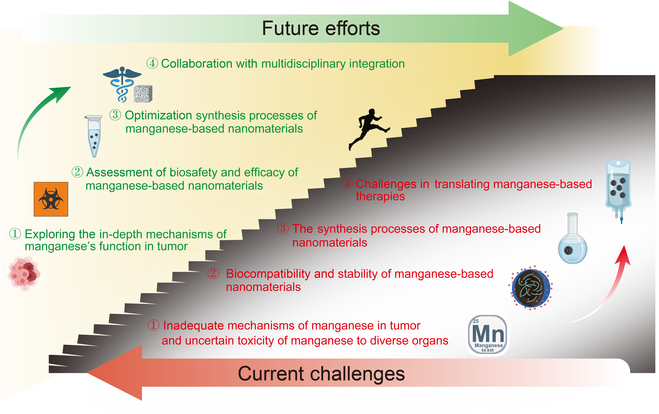
Roadmap of future efforts of Mn-based nanomaterials for clinical applications. Schematic illustration systematically summarizing the current limitations and clinical translational challenges associated with the application of Mn-based nanomaterials in tumor therapy, as well as outlining future research directions aimed at addressing these issues and promoting the development of Mn-based nanomaterials for clinical applications.

Facing the above-mentioned challenges of clinical application of Mn-based nanomaterials, future efforts can be made from mechanism exploration, material preparation, and performance evaluation. First, the exploration of the mechanism should focus on clarifying the toxicological effects of Mn on various organs of the human body and its safety measurement, while clarifying the effects of Mn on different tumors and expanding its possible molecular mechanism. Second, in order to improve the biocompatibility and stability of Mn-based nanomaterials, it is necessary to optimize the design of nanomaterials, which mainly includes controlling its particle size and shape, surface modification through biocompatible molecules such as polyethylene glycol, adding antibodies or ligands that can target tumors on the surface, and regulating the surface charge of nanomaterials to be neutral or negative [151,152]. The improvement of the synthesis process can improve its yield, reduce impurities and by-products, and reduce the impact of the synthesis process on the environment through the cooperation of multiple disciplines such as medicine, biology, and materials science. Last, there is a need to ensure that manganese-based nanomaterials comply with relevant safety standards and guidelines from development to clinical trials, in accordance with national and international regulatory authorities. There is also a need to establish a comprehensive toxicological evaluation system in preclinical studies; improve its blood compatibility, histocompatibility, immunogenicity, and pharmacokinetic evaluation; and further expand the evaluation of biosafety and efficacy in a variety of model organisms. It is also important to have an effective monitoring system to track the behavior of manganese-based nanomaterials in the human body and for timely patient feedback. With the rapid development of nanotechnology and further research, it is reasonable to believe that these problems will be gradually solved (Fig. 5). Accordingly, Mn-based nanomaterials are expected to become an important clinical cancer diagnosis and treatment method.

## Data Availability

No datasets were generated or analyzed during the current study.

## References

[1] Siegel RL, Giaquinto AN, Jemal A. Cancer statistics, 2024. CA Cancer J Clin. 2024;74(1):12–49. 38230766 10.3322/caac.21820

[2] Siegel RL, Miller KD, Wagle NS, Jemal A. Cancer statistics, 2023. CA Cancer J Clin. 2023;73(1):17–48. 36633525 10.3322/caac.21763

[3] Bray F, Laversanne M, Sung H, Ferlay J, Siegel RL, Soerjomataram I, Jemal A. Global cancer statistics 2022: GLOBOCAN estimates of incidence and mortality worldwide for 36 cancers in 185 countries. CA Cancer J Clin. 2024;74(3):229–263. 38572751 10.3322/caac.21834

[4] Sharifi M, Avadi MR, Attar F, Dashtestani F, Ghorchian H, Rezayat SM, Saboury AA, Falahati M. Cancer diagnosis using nanomaterials based electrochemical nanobiosensors. Biosens Bioelectron. 2019;126:773–784. 30554099 10.1016/j.bios.2018.11.026

[5] Liu Y, Niu R, Deng R, Wang Y, Song S, Zhang H. Multi-enzyme co-expressed nanomedicine for anti-metastasis tumor therapy by up-regulating cellular oxidative stress and depleting cholesterol. Adv Mater. 2024;36(2):e2307752. 37734072 10.1002/adma.202307752

[6] Jiang Z, Zhang W, Zhang J, Liu T, Xing J, Zhang H, Tang D. Nanomaterial-based drug delivery systems: A new weapon for cancer immunotherapy. Int J Nanomedicine. 2022;17:4677–4696. 36211025 10.2147/IJN.S376216 PMC9541303

[7] Liu Y, Li J, Chen M, Chen X, Zheng N. Palladium-based nanomaterials for cancer imaging and therapy. Theranostics. 2020;10(22):10057–10077. 32929334 10.7150/thno.45990 PMC7481408

[8] Cheng Z, Li M, Dey R, Chen Y. Nanomaterials for cancer therapy: Current progress and perspectives. J Hematol Oncol. 2021;14(1):85. 34059100 10.1186/s13045-021-01096-0 PMC8165984

[9] Siddiqui J, Taheri M, Ul Alam A, Deen MJ. Nanomaterials in smart packaging applications: A review. Small. 2022;18(1):2101171. 10.1002/smll.202101171 34514693

[10] Xin J, Deng C, Aras O, Zhou M, Wu C, An F. Chemodynamic nanomaterials for cancer theranostics. J Nanobiotechnol. 2021;19(1):192. 10.1186/s12951-021-00936-y PMC8240398 34183023

[11] Yu X-T, Sui S-Y, He Y-X, Yu C-H, Peng Q. Nanomaterials-based photosensitizers and delivery systems for photodynamic cancer therapy. Biomater Adv. 2022;135, 212725. 10.1016/j.bioadv.2022.212725 35929205

[12] Somvanshi SB, Jadhav SA, Gawali SS, Zakde K, Jadhav KM. Core-shell structured superparamagnetic Zn-mg ferrite nanoparticles for magnetic hyperthermia applications. J Alloys Compd. 2023;947, 169574.

[13] Kharat PB, Somvanshi SB, Khirade PP, Jadhav KM. Induction heating analysis of surface-functionalized nanoscale CoFe_2_O_4_ for magnetic fluid hyperthermia toward noninvasive cancer treatment. ACS Omega. 2020;5(36):23378–23384. 32954190 10.1021/acsomega.0c03332 PMC7496002

[14] Somvanshi SB, Patade SR, Andhare DD, Jadhav SA, Khedkar MV, Kharat PB, Khirade PP, Jadhav KM. Hyperthermic evaluation of oleic acid coated nano-spinel magnesium ferrite: Enhancement via hydrophobic-to-hydrophilic surface transformation. J Alloys Compd. 2020;835:155422.

[15] Gupta T, Pawar B, Vasdev N, Pawar V, Tekade RK. Carbonaceous nanomaterials for phototherapy of cancer. Technol Cancer Res Treat. 2023;22:15330338231186388. 37461375 10.1177/15330338231186388 PMC10357070

[16] Tang L, Zhang A, Zhang Z, Zhao Q, Li J, Mei Y, Yin Y, Wang W. Multifunctional inorganic nanomaterials for cancer photoimmunotherapy. Cancer Commun. 2022;42(2):141–163. 10.1002/cac2.12255 PMC8822595 35001556

[17] Lv M, Chen M, Zhang R, Zhang W, Wang C, Zhang Y, Wei X, Guan Y, Liu J, Feng K, et al. Manganese is critical for antitumor immune responses via cGAS-STING and improves the efficacy of clinical immunotherapy. Cell Res. 2020;30(11):966–979. 32839553 10.1038/s41422-020-00395-4 PMC7785004

[18] Zhang T, Hu C, Zhang W, Ruan Y, Ma Y, Chen D, Huang Y, Fan S, Lin W, Huang Y, et al. Advances of MnO_2_ nanomaterials as novel agonists for the development of cGAS-STING-mediated therapeutics. Front Immunol. 2023;14: 1156239. 10.3389/fimmu.2023.1156239 PMC10154562 37153576

[19] Lei H, Li Q, Li G, Wang T, Lv X, Pei Z, Gao X, Yang N, Gong F, Yang Y, et al. Manganese molybdate nanodots with dual amplification of STING activation for “cycle” treatment of metalloimmunotherapy. Bioactive Materials. 2024;31:53–62. 37601278 10.1016/j.bioactmat.2023.07.026 PMC10432900

[20] Pan S, Sun Z, Zhao B, Miao L, Zhou Q, Chen T, Zhu X, Chen T, Zhu X. Therapeutic application of manganese-based nanosystems in cancer radiotherapy. Biomaterials. 2023;302:122321. 37722183 10.1016/j.biomaterials.2023.122321

[21] Wu M, Liao Y, Guo D, Zhai M, Xia D, Zhang Z, Liu X, Huang Y. Manganese-based nanomaterials in diagnostics and chemodynamic therapy of cancers: New development. RSC Adv. 2024;14(21):14722–14741. 38716093 10.1039/d4ra01655f PMC11074770

[22] Zheng R, Guo J, Cai X, Bin L, Lu C, Singh A, Trivedi M, Kumar A, Liu J. Manganese complexes and manganese-based metal-organic frameworks as contrast agents in MRI and chemotherapeutics agents: Applications and prospects. Colloids Surf B Biointerfaces. 2022;213. 10.1016/j.colsurfb.2022.112432 35259704

[23] Liu Y, Zhao H, Wang S, Niu R, Bi S, Han W-K, Wang Y, Song S, Zhang H, Zhao Y. A Wurster-type covalent organic framework with internal electron transfer-enhanced catalytic capacity for tumor therapy. J Am Chem Soc. 2024;146(40):27345–27361. 39316459 10.1021/jacs.4c05555

[24] Huang Z, Huang S, Song S, Ding Y, Zhou H, Zhang S, Weng L, Zhang Y, Hu Y, Yuan A, et al. Two-dimensional coordination risedronate-manganese nanobelts as adjuvant for cancer radiotherapy and immunotherapy. Nat Commun. 2024;15(1):8692. 39375342 10.1038/s41467-024-53084-w PMC11458765

[25] Cao Y, Li Y, Ren C, Yang C, Hao R, Mu T. Manganese-based nanomaterials promote synergistic photo-immunotherapy: Green synthesis, underlying mechanisms, and multiple applications. J Mater Chem B. 2024;12(17):4097–4117. 38587869 10.1039/d3tb02844e

[26] Shi J-H, Chen Y-X, Feng Y, Yang X, Lin J, Wang T, Wei C-C, Ma X-H, Yang R, Cao D, et al. Fructose overconsumption impairs hepatic manganese homeostasis and ammonia disposal. Nat Commun. 2023;14(1):7934. 38040719 10.1038/s41467-023-43609-0 PMC10692208

[27] Martins AC, Krum BN, Queiros L, Tinkov AA, Skalny AV, Bowman AB, Aschner M. Manganese in the diet: Bioaccessibility, adequate intake, and neurotoxicological effects. J Agric Food Chem. 2020;68(46):12893–12903. 32298096 10.1021/acs.jafc.0c00641

[28] Foulquier F, Legrand D. Biometals and glycosylation in humans: Congenital disorders of glycosylation shed lights into the crucial role of Golgi manganese homeostasis. BBA-Gen Subjects. 2020;1864(10):129674. 10.1016/j.bbagen.2020.129674 32599014

[29] Kim GW, Lee DH, Jeon YH, Yoo J, Kim SY, Lee SW, Cho HY, Kwon SH. Glutamine synthetase as a therapeutic target for cancer treatment. Int J Mol Sci. 2021;22(4):1701. 33567690 10.3390/ijms22041701 PMC7915753

[30] Liziczai M, Fuchs A, Manatschal C, Dutzler R. Structural basis for metal ion transport by the human SLC11 proteins DMT1 and NRAMP1. Nat Commun. 2025;16(1):761. 39824808 10.1038/s41467-024-54705-0 PMC11742427

[31] Wang NEE, Courcelle EJJ, Coltman SM, Spolek RL, Courcelle J, Courcelle CT. Manganese transporters regulate the resumption of replication in hydrogen peroxide-stressed *Escherichia coli*. Biometals. 2023;36(6):1361–1376. 37493920 10.1007/s10534-023-00523-8 PMC12279252

[32] Erikson KM, Aschner M, Manganese: Its role in disease and health. In: Carver PL, editor. *Essential metals in medicine: Therapeutic use and toxicity of metal ions in the clinic.* Berlin, Boston: De Gruyter; 2019. Vol. 19, p. 253–266.

[33] Chen X, Zhang L, Zeng H, Meng W, Liu G, Zhang W, Zhao P, Zhang Q, Chen M, Chen J. Manganese-based immunomodulatory nanocomposite with catalase-like activity and microwave-enhanced ROS elimination ability for efficient rheumatoid arthritis therapy. Small. 2023;19(50): Article e2304610. 37632302 10.1002/smll.202304610

[34] Wang H, Zhang S, Yang F, Xin R, Wang S, Cui D, Sun Y. The gut microbiota confers protection in the CNS against neurodegeneration induced by manganism. Biomed Pharmacother. 2020;127:110150. 32330797 10.1016/j.biopha.2020.110150

[35] Eshak ES, Muraki I, Imano H, Yamagishi K, Tamakoshi A, Iso H. Manganese intake from foods and beverages is associated with a reduced risk of type 2 diabetes. Maturitas. 2021;143:127–131. 33308618 10.1016/j.maturitas.2020.10.009

[36] Cheng G, Wu J, Ji M, Hu W, Wu C, Jiang J. TET2 inhibits the proliferation and metastasis of lung adenocarcinoma cells via activation of the cGAS-STING signalling pathway. BMC Cancer. 2023;23(1):825. 37667220 10.1186/s12885-023-11343-x PMC10478367

[37] Li W, Lu L, Lu J, Wang X, Yang C, Jin J, Wu L, Hong X, Li F, Cao D, et al. cGAS-STING-mediated DNA sensing maintains CD8^+^ T cell stemness and promotes antitumor T cell therapy. Sci Transl Med. 2020;12(549): Article eaay9013. 32581136 10.1126/scitranslmed.aay9013

[38] Hooy RM, Massaccesi G, Rousseau KE, Chattergoon MA, Sohn J. Allosteric coupling between Mn^2+^ and dsDNA controls the catalytic efficiency and fidelity of cGAS. Nucleic Acids Res. 2020;48(8):4435–4447. 32170294 10.1093/nar/gkaa084 PMC7192592

[39] Zhang R, Wang C, Guan Y, Wei X, Sha M, Yi M, Jing M, Lv M, Guo W, Xu J, et al. Manganese salts function as potent adjuvants. Cell Mol Immunol. 2021;18(5):1222–1234. 33767434 10.1038/s41423-021-00669-w PMC8093200

[40] Gu Y, Tang J, Zhang F, Qu Y, Zhao M, Li M, Xie Z, Wang X, Song L, Jiang Z, et al. Manganese potentiates lipopolysaccharide-induced innate immune responses and septic shock. Int J Biol Macromol. 2023;230:123202. 36639076 10.1016/j.ijbiomac.2023.123202

[41] Huang S, Gao Y, Li H, Wang R, Zhang X, Wang X, Huang D, Zhang L, Santos HA, Yin Z, et al. Manganese@albumin nanocomplex and its assembled nanowire activate TLR4-dependent signaling cascades of macrophages. Adv Mater. 2024;36(5): Article e2310979. 37994277 10.1002/adma.202310979

[42] Abu-Elfotuh K, Hamdan AME, Mohammed AA, Atwa AM, Kozman MR, Ibrahim AM, Motawea SM, Selim HMRM, Tohamy STK, El-Din MN, et al. Neuroprotective effects of some nutraceuticals against manganese-induced Parkinson’s disease in rats: Possible modulatory effects on TLR4/NLRP3/NF-κB, GSK-3β, Nrf2/HO-1, and apoptotic pathways. Pharmaceuticals. 2022;15(12):1554. 36559006 10.3390/ph15121554 PMC9785377

[43] Wang Y, Liu T, Li X, Sheng H, Ma X, Hao L. Ferroptosis-inducing nanomedicine for cancer therapy. Front Pharmacol. 2021;12:735965. 34987385 10.3389/fphar.2021.735965 PMC8722674

[44] Zhang S, Kang L, Dai X, Chen J, Chen Z, Wang M, Jiang H, Wang X, Bu S, Liu X, et al. Manganese induces tumor cell ferroptosis through type-I IFN dependent inhibition of mitochondrial dihydroorotate dehydrogenase. Free Radic Biol Med. 2022;193(Pt 1):202–212. 36228830 10.1016/j.freeradbiomed.2022.10.004

[45] Cheng J, Zhu Y, Xing X, Xiao J, Chen H, Zhang H, Wang D, Zhang Y, Zhang G, Wu Z, et al. Manganese-deposited iron oxide promotes tumor-responsive ferroptosis that synergizes the apoptosis of cisplatin. Theranostics. 2021;11(11):5418–5429. 33859755 10.7150/thno.53346 PMC8039957

[46] Tang H, Chen D, Li C, Zheng C, Wu X, Zhang Y, Song Q, Fei W. Dual GSH-exhausting sorafenib loaded manganese-silica nanodrugs for inducing the ferroptosis of hepatocellular carcinoma cells. Int J Pharm. 2019;572:118782. 31678528 10.1016/j.ijpharm.2019.118782

[47] Wang C, Guan Y, Lv M, Zhang R, Guo Z, Wei X, Du X, Yang J, Li T, Wan Y, et al. Manganese increases the sensitivity of the cGAS-STING pathway for double-stranded DNA and is required for the host defense against DNA viruses. Immunity. 2018;48(4):675–687.e7. 29653696 10.1016/j.immuni.2018.03.017

[48] Wang S, Li F, Qiao R, Hu X, Liao H, Chen L, Wu J, Wu H, Zhao M, Liu J, et al. Arginine-rich manganese silicate nanobubbles as a ferroptosis-inducing agent for tumor-targeted theranostics. ACS Nano. 2018;12(12):12380–12392. 30495919 10.1021/acsnano.8b06399

[49] Tinkov AA, Nguyen TT, Santamaria A, Bowman AB, Buha Djordjevic A, Paoliello MMB, Skalny AV, Aschner M. Sirtuins as molecular targets, mediators, and protective agents in metal-induced toxicity. Arch Toxicol. 2021;95(7):2263–2278. 34028595 10.1007/s00204-021-03048-6

[50] Wang J, Qu C, Shao X, Song G, Sun J, Shi D, Jia R, An H, Wang H. Carrier-free nanoprodrug for p53-mutated tumor therapy via concurrent delivery of zinc-manganese dual ions and ROS. Bioact Mater. 2023;20:404–417. 35784636 10.1016/j.bioactmat.2022.06.005 PMC9218170

[51] Tidball AM, Bryan MR, Uhouse MA, Kumar KK, Aboud AA, Feist JE, Ess KC, Neely MD, Aschner M, Bowman AB. A novel manganese-dependent ATM-p53 signaling pathway is selectively impaired in patient-based neuroprogenitor and murine striatal models of Huntington’s disease. Hum Mol Genet. 2015;24(7):1929–1944. 25489053 10.1093/hmg/ddu609 PMC4355025

[52] Liu J, Guo W, Li J, Geng J, Chen Q, Gao J. Tumor-targeting novel manganese complex induces ROS-mediated apoptotic and autophagic cancer cell death. Int J Mol Med. 2015;35(3):607–616. 25604962 10.3892/ijmm.2015.2073 PMC4314420

[53] Nicastro R, Gaillard H, Zarzuela L, Peli-Gulli M-P, Fernandez-Garcia E, Tome M, Garcia-Rodriguez N, Duran RV, De Virgilio C, Wellinger RE. Manganese is a physiologically relevant TORC1 activator in yeast and mammals. elife. 2022;11:e80497. 35904415 10.7554/eLife.80497 PMC9337852

[54] Bo L-Y, Li T-J, Zhao X-H. Copper or manganese supplementation endows the peptic hydrolysate from bovine lactoferrin with enhanced activity to human gastric cancer AGS cells. Biol Trace Elem Res. 2019;189(1):64–74. 30069694 10.1007/s12011-018-1468-x

[55] Bryan MR, Nordham KD, Rose DIR, O’Brien MT, Joshi P, Foshage AM, Goncalves FM, Nitin R, Uhouse MA, Aschner M, et al. Manganese acts upon insulin/IGF receptors to phosphorylate AKT and increase glucose uptake in Huntington’s disease cells. Mol Neurobiol. 2020;57(3):1570–1593. 31797328 10.1007/s12035-019-01824-1 PMC7062569

[56] Bryan MR, Bowman AB. Manganese and the insulin-IGF signaling network in Huntington’s disease and other neurodegenerative disorders. Adv Neurobiol. 2017;18:113–142. 28889265 10.1007/978-3-319-60189-2_6 PMC6559248

[57] Slordahl TS, Hov H, Holt RU, Baykov V, Syversen T, Sundan A, Waage A, Borset M. Mn^2+^ regulates myeloma cell adhesion differently than the proadhesive cytokines HGF, IGF-1, and SDF-1. Eur J Haematol. 2008;81(6):437–447. 18774952 10.1111/j.1600-0609.2008.01148.x

[58] Tong S, Yu Z, Yin F, Yang Q, Chu J, Huang L, Gao W, Qian M. Manganese-based Prussian blue nanoparticles inhibit tumor proliferation and migration via the MAPK pathway in pancreatic cancer. Front Chem. 2022;10:1026924. 36353142 10.3389/fchem.2022.1026924 PMC9638070

[59] Liang S, Li J, Zou Z, Mao M, Ming S, Lin F, Zhang Z, Cao C, Zhou J, Zhang Y, et al. Tetrahedral DNA nanostructures synergize with MnO_2_ to enhance antitumor immunity via promoting STING activation and M1 polarization. Acta Pharm Sin B. 2022;12(5):2494–2505. 35646524 10.1016/j.apsb.2021.12.010 PMC9136606

[60] Barber GN. STING: Infection, inflammation and cancer. Nat Rev Immunol. 2015;15(12):760–770. 26603901 10.1038/nri3921 PMC5004891

[61] Hanna C Jr, Willman M, Cole D, Mehkri Y, Liu S, Willman J, Lucke-Wold B. Review of meningioma diagnosis and management. Egypt J Neurosurg. 2023;38(1):16.

[62] Tian J, Wang J, Li S. Advances in the treatment of solid tumors in children and adolescents. Cancer Innov. 2023;2(2):131–139. 38090056 10.1002/cai2.66 PMC10686120

[63] Wu Y, Pan X, Xie H, Que L, Tang X. Research progress of biomineralization for the diagnosis and treatment of malignant tumors. Front Pharmacol. 2023;14:1335019. 38155903 10.3389/fphar.2023.1335019 PMC10752927

[64] Jiang Y, Zhao J, Zhang D. Manganese dioxide-based nanomaterials for medical applications. ACS Biomater Sci Eng. 2024;10(5):2680–2702. 38588342 10.1021/acsbiomaterials.3c01852

[65] Fan H, Guo Z. Tumor microenvironment-responsive manganese-based nanomaterials for cancer treatment. Coord Chem Rev. 2023;480:215027.

[66] Haque S, Tripathy S, Patra CR. Manganese-based advanced nanoparticles for biomedical applications: Future opportunity and challenges. Nanoscale. 2021;13(39):16405–16426. 34586121 10.1039/d1nr04964j

[67] Liu X, Rong P. Recent advances of manganese-based hybrid nanomaterials for cancer precision medicine. Front Oncol. 2021;11:707618. 34722253 10.3389/fonc.2021.707618 PMC8548572

[68] Zhang J, Xu L, Hu H, Chen E. The combination of MnO_2_@Lipo-coated gefitinib and bevacizumab inhibits the development of non-small cell lung cancer. Drug Deliv. 2022;29(1):466–477. 35147070 10.1080/10717544.2022.2032872 PMC8843201

[69] Yang H, Lu W-L, Huang T, Chen Q-Y, Gao J, Zhao Y. An aptamer-Fe^3+^ modified nanoparticle for lactate oxidation and tumor photodynamic therapy. Colloids Surf B Biointerfaces. 2018;164:192–200. 29413596 10.1016/j.colsurfb.2018.01.045

[70] Brito B, Ruggiero MR, Price TW, da Costa SM, Genicio N, Wilson AJ, Tyurina O, Rosecker V, Eykyn TR, Banobre-Lopez M, et al. Redox double-switch cancer theranostics through Pt(IV) functionalised manganese dioxide nanostructures. Nanoscale. 2023;15(25):10763–10775. 37325846 10.1039/d3nr00076a PMC10311465

[71] Li M, Deng L, Li J, Yuan W, Gao X, Ni J, Jiang H, Zeng J, Ren J, Wang P. Actively targeted magnetothermally responsive nanocarriers/doxorubicin for thermochemotherapy of hepatoma. ACS Appl Mater Interfaces. 2018;10(48):41107–41117. 30403475 10.1021/acsami.8b14972

[72] Huang D, Xu D, Chen W, Wu R, Wen Y, Liu A, Lin L, Lin X, Wang X. Fe-MnO_2_ nanosheets loading dihydroartemisinin for ferroptosis and immunotherapy. Biomed Pharmacother. 2023;161:114431. 36827713 10.1016/j.biopha.2023.114431

[73] Liu X, Kifle MT, Xie H, Xu L, Luo M, Li Y, Huang Z, Gong Y, Wu Y, Xie C. Biomineralized manganese oxide nanoparticles synergistically relieve tumor hypoxia and activate immune response with radiotherapy in non-small cell lung cancer. Nano. 2022;12(18):3138. 10.3390/nano12183138 PMC9501587 36144927

[74] Ye LF, Chaudhary KR, Zandkarimi F, Harken AD, Kinslow CJ, Upadhyayula PS, Dovas A, Higgins DM, Tan H, Zhang Y, et al. Radiation-induced lipid peroxidation triggers ferroptosis and synergizes with ferroptosis inducers. ACS Chem Biol. 2020;15(2):469–484. 31899616 10.1021/acschembio.9b00939 PMC7180072

[75] Lang X, Green MD, Wang W, Yu J, Choi JE, Jiang L, Liao P, Zhou J, Zhang Q, Dow A, et al. Radiotherapy and immunotherapy promote tumoral lipid oxidation and ferroptosis via synergistic repression of SLC7A11. Cancer Discov. 2019;9(12):1673–1685. 31554642 10.1158/2159-8290.CD-19-0338 PMC6891128

[76] Zheng S, Hu H, Hou M, Zhu K, Wu Z, Qi L, Xia H, Liu G, Ren Y, Xu Y, et al. Proton pump inhibitor-enhanced nanocatalytic ferroptosis induction for stimuli-responsive dual-modal molecular imaging guided cancer radiosensitization. Acta Biomater. 2023;162:72–84. 36931419 10.1016/j.actbio.2023.03.011

[77] Chen W, Yan Y, Han R, Hu J, Hou Y, Tang K. Biodegradable flower-like manganese for synergistic photothermal and photodynamic therapy applications. Photochem Photobiol Sci. 2021;20(1):153–160. 33721245 10.1007/s43630-020-00010-w

[78] Xiang H, Xue F, Yi T, Tham HP, Liu J-G, Zhao Y. Cu_2-x_S nanocrystals cross-linked with chlorin e6-functionalized polyethylenimine for synergistic photodynamic and photothermal therapy of cancer. ACS Appl Mater Interfaces. 2018;10(19):16344–16351. 29697957 10.1021/acsami.8b04779

[79] Ding D, Guo W, Guo C, Sun J, Zheng N, Wang F, Yan M, Liu S. MoO_3-x_ quantum dots for photoacoustic imaging guided photothermal/photodynamic cancer treatment. Nanoscale. 2017;9(5):2020–2029. 28106206 10.1039/c6nr09046j

[80] Dash BS, Lu Y-J, Chen J-P. Enhancing photothermal/photodynamic therapy for glioblastoma by tumor hypoxia alleviation and heat shock protein inhibition using IR820-conjugated reduced graphene oxide quantum dots. ACS Appl Mater Interfaces. 2024;16(11):13543–13562. 38452225 10.1021/acsami.3c19152

[81] Chang R, Zou Q, Zhao L, Liu Y, Xing R, Yan X. Amino-acid-encoded supramolecular photothermal nanomedicine for enhanced cancer therapy. Adv Mater. 2022;34(16): Article e2200139. 35178775 10.1002/adma.202200139

[82] Zou Q, Bao J, Yan X. Functional nanomaterials based on self-assembly of endogenic NIR-absorbing pigments for diagnostic and therapeutic applications. Small Methods. 2022;6(4). 10.1002/smtd.202101359 35142112

[83] Khan HA, Lee Y-k, Shaik MR, Siddiqi NJ, Siddiqui MR, Alrashood ST, Alharbi AS, Ekhzaimy AA. Hybrid nanoparticles of manganese oxide and highly reduced graphene oxide for photodynamic therapy. Front Biosci. 2023;28(1): Article e2101359. 10.31083/j.fbl2801019 36722275

[84] Wang Y-Y, Wang W-L, Shen X-C, Zhou B, Chen T, Guo Z-X, Wen C-C, Jiang B-P, Liang H. Combination-responsive MoO_3-x_-hybridized hyaluronic acid hollow nanospheres for cancer phototheranostics. ACS Appl Mater Interfaces. 2018;10(49):42088–42101. 30408413 10.1021/acsami.8b15818

[85] Yin Q, Tan L, Lang Q, Ke X, Bai L, Guo K, Qiao R, Bai S. Plasmonic molybdenum oxide nanosheets supported silver nanocubes for enhanced near-infrared antibacterial activity: Synergism of photothermal effect, silver release and photocatalytic reactions. Appl Catal B Environ. 2018;224:671–680.

[86] Wu F, Zhang Q, Sun B, Chu X, Zhang M, She Z, Li Z, Zhou N, Wang J, Li A. MoO_3-x_ nanosheets-based platform for single NIR laser induced efficient PDT/PTT of cancer. J Control Release. 2021;338:46–55. 34391835 10.1016/j.jconrel.2021.08.022

[87] Liu Y, Zhen W, Jin L, Zhang S, Sun G, Zhang T, Xu X, Song S, Wang Y, Liu J, et al. All-in-one theranostic nanoagent with enhanced reactive oxygen species generation and modulating tumor microenvironment ability for effective tumor eradication. ACS Nano. 2018;12(5):4886–4893. 29727164 10.1021/acsnano.8b01893

[88] Wu F, Zhang M, Lu H, Liang D, Huang Y, Xia Y, Hu Y, Hu S, Wang J, Yi X, et al. Triple stimuli-responsive magnetic hollow porous carbon-based nanodrug delivery system for magnetic resonance imaging-guided synergistic photothermal/chemotherapy of cancer. ACS Appl Mater Interfaces. 2018;10(26):21939–21949. 29893126 10.1021/acsami.8b07213

[89] Kaidar-Person O, Meattini I, Poortmans P. Radiation therapy after breast conserving surgery increases long-term breast conservation for DCIS patients. Breast. 2018;37:179–180. 29100639 10.1016/j.breast.2017.10.013

[90] Ye JC, Formenti SC. Integration of radiation and immunotherapy in breast cancer—Treatment implications. Breast. 2018;38:66–74. 29253718 10.1016/j.breast.2017.12.005

[91] Filomeni G, Cerchiaro G, Da Costa Ferreira AM, De Martino A, Pedersen JZ, Rotilio G, Ciriolo MR. Pro-apoptotic activity of novel Isatin-Schiff base copper(II) complexes depends on oxidative stress induction and organelle-selective damage. J Biol Chem. 2007;282(16):12010–12021. 17327230 10.1074/jbc.M610927200

[92] Yadamani S, Neamati A, Homayouni-Tabrizi M, Beyramabadi SA, Yadamani S, Gharib A, Morsali A, Khashi M. Treatment of the breast cancer by using low frequency electromagnetic fields and Mn(II) complex of a Schiff base derived from the pyridoxal. Breast. 2018;41:107–112. 30025273 10.1016/j.breast.2018.07.001

[93] Patade SR, Andhare DD, Somvanshi SB, Jadhav SA, Khedkar MV, Jadhav KM. Self-heating evaluation of superparamagnetic MnFe_2_O_4_ nanoparticles for magnetic fluid hyperthermia application towards cancer treatment. Ceram Int. 2020;46(16):25576–25583.

[94] Somvanshi SB, Kharat PB, Khedkar MV, Jadhav KM. Hydrophobic to hydrophilic surface transformation of nano-scale zinc ferrite via oleic acid coating: Magnetic hyperthermia study towards biomedical applications. Ceram Int. 2020;46(6):7642–7653.

[95] Phalake SS, Somvanshi SB, Tofail SAM, Thorat ND, Khot VM. Functionalized manganese iron oxide nanoparticles: A dual potential magneto-chemotherapeutic cargo in a 3D breast cancer model. Nanoscale. 2023;15(38):15686–15699. 37724853 10.1039/d3nr02816j

[96] Xu Y, Tan W, Chen M, Chen S, Tang K, Liao H, Niu C. MnO_2_ coated multi-layer nanoplatform for enhanced sonodynamic therapy and MR imaging of breast cancer. Front Bioeng Biotechnol. 2022;10:955127. 36338124 10.3389/fbioe.2022.955127 PMC9627152

[97] Zhao Y, Huang T, Wang S, Yao S, Hu Q, Wan X, Guo N, Zhang Y, Li L. Manganese oxide-modified bismuth oxychloride piezoelectric nanoplatform with multiple enzyme-like activities for cancer sonodynamic therapy. J Colloid Interface Sci. 2023;640:839–850. 36905893 10.1016/j.jcis.2023.03.008

[98] Zhang S, Xia S, Chen L, Chen Y, Zhou J. Covalent organic framework nanobowls as activatable nanosensitizers for tumor-specific and ferroptosis-augmented sonodynamic therapy. Adv Sci. 2023;10(6):e2206009. 10.1002/advs.202206009 PMC9951320 36594611

[99] Sun S, Chen Q, Tang Z, Liu C, Li Z, Wu A, Lin H. Tumor microenvironment stimuli-responsive fluorescence imaging and synergistic cancer therapy by carbon-dot-Cu^2+^ nanoassemblies. Angew Chem-Int Ed. 2020;59(47):21041–21048. 10.1002/anie.202007786 32914924

[100] Fu S, Yang R, Ren J, Liu J, Zhang L, Xu Z, Kang Y, Xue P. Catalytically active CoFe_2_O_4_ nanoflowers for augmented sonodynamic and chemodynamic combination therapy with elicitation of robust immune response. ACS Nano. 2021;15(7):11953–11969. 34142808 10.1021/acsnano.1c03128

[101] Gao F, Sun M, Zhang J, Chang Y, Gao W, Ma G, Ma X, Guo Y. Fenton-like reaction and glutathione depletion by chiral manganese dioxide nanoparticles for enhanced chemodynamic therapy and chemotherapy. J Colloid Interface Sci. 2022;616:369–378. 35220185 10.1016/j.jcis.2022.02.060

[102] Qiao W, Chen J, Zhou H, Hu C, Dalangood S, Li H, Yang D, Yang Y, Gui J. A single-atom manganese nanozyme Mn-N/C promotes anti-tumor immune response via eliciting type I interferon signaling. Adv Sci. 2024;11(14): Article e2305979. 10.1002/advs.202305979 PMC11005736 38308189

[103] Li Z, Chu Z, Yang J, Qian H, Xu J, Chen B, Tian T, Chen H, Xu Y, Wang F. Immunogenic cell death augmented by manganese zinc sulfide nanoparticles for metastatic melanoma immunotherapy. ACS Nano. 2022;16(9):15471–15483. 35981098 10.1021/acsnano.2c08013

[104] Oliveira G, Wu CJJ. Dynamics and specificities of T cells in cancer immunotherapy. Nat Rev Cancer. 2023;23(5):295–316. 37046001 10.1038/s41568-023-00560-y PMC10773171

[105] Li Z, Pai R, Gupta S, Currenti J, Guo W, Di Bartolomeo A, Feng H, Zhang Z, Li Z, Liu L, et al. Presence of onco-fetal neighborhoods in hepatocellular carcinoma is associated with relapse and response to immunotherapy. Nat Cancer. 2024;5(1):167–186. 38168935 10.1038/s43018-023-00672-2

[106] Heymann M-F, Schiavone K, Heymann D. Bone sarcomas in the immunotherapy era. Br J Pharmacol. 2021;178(9):1955–1972. 31975481 10.1111/bph.14999

[107] Binnewies M, Roberts EW, Kersten K, Chan V, Fearon DF, Merad M, Coussens LM, Gabrilovich DI, Ostrand-Rosenberg S, Hedrick CC, et al. Understanding the tumor immune microenvironment (TIME) for effective therapy. Nat Med. 2018;24(5):541–550. 29686425 10.1038/s41591-018-0014-x PMC5998822

[108] Ding B, Chen H, Tan J, Meng Q, Zheng P, Pa M, Lin J. ZIF-8 nanoparticles evoke pyroptosis for high-efficiency cancer immunotherapy. Angew Chem Int Ed. 2023;62(10): Article e202215307. 10.1002/anie.202215307 36629270

[109] Niu R, Liu Y, Xu B, Deng R, Zhou S, Cao Y, Li W, Zhang H, Zheng H, Song S, et al. Programmed targeting pyruvate metabolism therapy amplified single-atom nanozyme-activated pyroptosis for immunotherapy. Adv Mater. 2024;36(24): Article e2312124. 38314930 10.1002/adma.202312124

[110] Cheng L, Zhang P, Liu Y, Liu Z, Tang J, Xu L, Liu J. Multifunctional hybrid exosomes enhanced cancer chemo-immunotherapy by activating the STING pathway. Biomaterials. 2023;301:122259. 37531777 10.1016/j.biomaterials.2023.122259

[111] Luo S, Yang Y, Chen L, Kannan PR, Yang W, Zhang Y, Zhao R, Liu X, Li Y, Kong X. Outer membrane vesicle-wrapped manganese nanoreactor for augmenting cancer metalloimmunotherapy through hypoxia attenuation and immune stimulation. Acta Biomater. 2024;181:402–414. 38734282 10.1016/j.actbio.2024.05.010

[112] Wang P, Wang Y, Li H, Wang M, Wang Y, Wang X, Ran L, Xin H, Ma J, Tian G, et al. A homologous-targeting cGAS-STING agonist multimodally activates dendritic cells for enhanced cancer immunotherapy. Acta Biomater. 2024;177:400–413. 38336268 10.1016/j.actbio.2024.02.003

[113] Du Q, Luo Y, Xu L, Du C, Zhang W, Xu J, Liu Y, Liu B, Chen S, Wang Y, et al. Smart responsive Fe/Mn nanovaccine triggers liver cancer immunotherapy via pyroptosis and pyroptosis-boosted cGAS-STING activation. J Nanobiotechnol. 2024;22(1):95. 10.1186/s12951-024-02354-2 PMC10918897 38448959

[114] Zhang J, Yuan B, Zhang H, Li H. Human epithelial ovarian cancer cells expressing CD105, CD44 and CD106 surface markers exhibit increased invasive capacity and drug resistance. Oncol Lett. 2019;17(6):5351–5360. 31186752 10.3892/ol.2019.10221 PMC6507388

[115] Guo T, Zhu Y, Yue M, Wang F, Li Z, Lin M. The therapeutic effects of DDP/CD44-shRNA nanoliposomes in AMF on ovarian cancer. Front Oncol. 2022;12:811783. 35402279 10.3389/fonc.2022.811783 PMC8989969

[116] Zhang Z, Zhang Y, Xia S, Kong Q, Li S, Liu X, Junqueira C, Meza-Sosa KF, Mok TMY, Ansara J, et al. Gasdermin E suppresses tumour growth by activating anti-tumour immunity. Nature. 2020;579(7799):415–420. 32188940 10.1038/s41586-020-2071-9 PMC7123794

[117] Zhong H, Chen G, Li T, Huang J, Lin M, Li B, Xiao Z, Shuai X. Nanodrug augmenting antitumor immunity for enhanced TNBC therapy via pyroptosis and cGAS-STING activation. Nano Lett. 2023;23(11):5083–5091. 37220198 10.1021/acs.nanolett.3c01008

[118] Yu J, Cheng Z-G, Han Z-Y, Liu F-Y, Zheng R-Q, Cheng W, Wei Q, Yu S-Y, Li Q-y, He GZ, et al. Period-dependent survival benefit of percutaneous microwave ablation for hepatocellular carcinoma: A 12-year real-world, multicentric experience. Liver Cancer. 2022;11(4):341–353. 35978603 10.1159/000522134 PMC9294937

[119] Wei Y, Niu W-Q, Zhao Z-L, Wu J, Peng L-L, Li Y, Yu M-A. Microwave ablation versus surgical resection for solitary T1N0M0 papillary thyroid carcinoma. Radiology. 2022;304(3):704–713. 35536133 10.1148/radiol.212313

[120] Zhou Y, Shu G, Luo Y, Wang F, Jing X, Pan J, Sun S-K. Achieving complete tumor clearance: A minimalist manganese hydrogel for magnetic resonance imaging-guided synergetic microwave ablation and chemodynamic therapy. Adv Healthc Mater. 2024;13(9): Article e2303268. 38140916 10.1002/adhm.202303268

[121] Kalaiselvan CR, Laha SS, Somvanshi SB, Tabish TA, Thorat ND, Sahu NK. Manganese ferrite (MnFe_2_O_4_) nanostructures for cancer theranostics. Coord Chem Rev. 2022;473:214809.

[122] Yang G, Xia J, Dai X, Zhao H, Gao W, Ding W, Tao X, Zhu L. A targeted multi-crystalline manganese oxide as a tumor-selective nano-sized MRI contrast agent for early and accurate diagnosis of tumors. Int J Nanomedicine. 2024;19:527–540. 38260241 10.2147/IJN.S444061 PMC10802178

[123] Lv Y, Kan J, Luo M, Yang C, Luo X, Lin X, Li H, Li X, Li Y, Yang C, et al. Multifunctional nanosnowflakes for T1-T2 double-contrast enhanced MRI and PAI guided oxygen self-supplementing effective anti-tumor therapy. Int J Nanomedicine. 2022;17:4619–4638. 36211026 10.2147/IJN.S379526 PMC9533148

[124] Guan G, Liu H, Xu J, Zhang Q, Dong Z, Lei L, Zhang C, Yue R, Gao H, Song G, et al. Ultrasmall PtMn nanoparticles as sensitive manganese release modulator for specificity cancer theranostics. J Nanobiotechnol. 2023;21(1):434. 10.1186/s12951-023-02172-y PMC10657629 37980476

[125] Gowtham P, Girigoswami K, Pallavi P, Harini K, Gurubharath I, Girigoswami A. Alginate-derivative encapsulated carbon coated manganese-ferrite Nanodots for multimodal medical imaging. Pharmaceutics. 2022;14(12):2550. 36559045 10.3390/pharmaceutics14122550 PMC9782169

[126] An Y, Chen W, Li Y, Zhao H, Ye D, Liu H, Wu K, Ju H. Crosslinked albumin-manganese nanoaggregates with sensitized T_1_ relaxivity and indocyanine green loading for multimodal imaging and cancer phototherapy. J Mater Chem B. 2023;11(10):2157–2165. 36779282 10.1039/d2tb02529a

[127] Cui M, Tang Z, Ahmad Z, Pan C, Lu Y, Ali K, Huang S, Lin X, Wahab A, Iqbal MZ, et al. Facile synthesis of manganese-hafnium nanocomposites for multimodal MRI/CT imaging and in vitro photodynamic therapy of colon cancer. Colloids Surf B Biointerfaces. 2024;237: Article 113834. 38479259 10.1016/j.colsurfb.2024.113834

[128] Hu D, Xia M, Wu L, Liu H, Chen Z, Xu H, He C, Wen J, Xu X. Challenges and advances for glioma therapy based on inorganic nanoparticles. Materials Today Bio. 2023;20:100673. 10.1016/j.mtbio.2023.100673 PMC10333687 37441136

[129] Shao Y, Yang G, Lin J, Fan X, Guo Y, Zhu W, Cai Y, Huang H, Hu D, Pang W, et al. Shining light on chiral inorganic nanomaterials for biological issues. Theranostics. 2021;11(19):9262–9295. 34646370 10.7150/thno.64511 PMC8490512

[130] Li X, Wang Q, Yu S, Zhang M, Liu X, Deng G, Liu Y, Wu S. Multifunctional MnO_2_-based nanoplatform-induced ferroptosis and apoptosis for synergetic chemoradiotherapy. Nanomedicine. 2021;16(26):2343–2361. 34523352 10.2217/nnm-2021-0286

[131] Deng Z, Xi M, Zhang C, Wu X, Li Q, Wang C, Fang H, Sun G, Zhang Y, Yang G, et al. Biomineralized MnO_2_ nanoplatforms mediated delivery of immune checkpoint inhibitors with STING pathway activation to potentiate cancer radio-immunotherapy. ACS Nano. 2023;17(5):4495–4506. 36848115 10.1021/acsnano.2c10352

[132] Feng Y, Wang G, Li W, Yan J, Yu X, Tian H, Li B, Dai Y. PhotoPyro-induced cGAS-STING pathway activation enhanced anti-melanoma immunotherapy via a manganese-coordinated nanomedicine. Adv Healthc Mater. 2024;13(6): Article e2302811. 37909376 10.1002/adhm.202302811

[133] Lu H, Li W, Qiu P, Zhang X, Qin J, Cai Y, Lu X. MnO_2_ doped graphene nanosheets for carotid body tumor combination therapy. Nanoscale Adv. 2022;4(20):4304–4313. 36321141 10.1039/d2na00086e PMC9552922

[134] Chen X, Li Q, Huang Z, Lin W, Ma Y. Construction and evaluation of curcumin upconversion nanocarriers decorated with MnO_2_ for tumor photodynamic therapy. Drug Deliv Transl Res. 2022;12(11):2678–2692. 35061221 10.1007/s13346-022-01118-5

[135] Wang L, Wu M, Pan Y, Xie D, Hong C, Li J, Ma X, Xu H, Li H, Chen T, et al. Sequential targeting biomimetic nano platform for enhanced mild photothermal therapy and chemotherapy of tumor. Comput Struct Biotechnol J. 2023;21:2780–2791. 37181660 10.1016/j.csbj.2023.04.024 PMC10172638

[136] Guo J, Zhao W, Xiao X, Liu S, Liu L, Zhang L, Li L, Li Z, Li Z, Xu M, et al. Reprogramming exosomes for immunity-remodeled photodynamic therapy against non-small cell lung cancer. Bioact Mater. 2024;39:206–223. 38827172 10.1016/j.bioactmat.2024.05.030 PMC11141154

[137] Tao W, Wang N, Ruan J, Cheng X, Fan L, Zhang P, Lu C, Hu Y, Che C, Sun D, et al. Enhanced ROS-boosted phototherapy against pancreatic cancer via Nrf2-mediated stress-defense pathway suppression and Ferroptosis induction. ACS Appl Mater Interfaces. 2022;14(5):6404–6416. 35077153 10.1021/acsami.1c22861

[138] Yin P, Sun D, Deng Y, Zhu X, Wang Y, Yang J, Feng X. Metal-organic cage as a theranostic nanoplatform for magnetic resonance imaging guided chemodynamic therapy. Theranostics. 2024;14(12):4861–4873. 39239515 10.7150/thno.97264 PMC11373615

[139] Su M, Chen Y, Jia L, Zhang Z. Camptothecin-loaded and manganese dioxide-coated polydopamine nanomedicine used for magnetic resonance imaging diagnosis and chemo-photothermal therapy for lung cancer. Int J Nanomedicine. 2022;17:6687–6705. 36597434 10.2147/IJN.S359300 PMC9805739

[140] Qiao N, Wang H, Xu Y, Chang Y, Xie M, Bai S, He C, Qin M, Zhong X, Jiang M, et al. A Mn-Al double adjuvant nanovaccine to induce strong humoral and cellular immune responses. J Control Release. 2023;358:190–203. 37116543 10.1016/j.jconrel.2023.04.036

[141] He Q, Zheng R, Ma J, Zhao L, Shi Y, Qiu J. Responsive manganese-based nanoplatform amplifying cGAS-STING activation for immunotherapy. Biomater Res. 2023;27(1):29. 37061706 10.1186/s40824-023-00374-x PMC10105937

[142] Du S, Chen C, Qu S, Song H, Yang J, Li Y, Liu K, Lu Q, Luo W, Wang R, et al. DNAzyme-assisted nano-herb delivery system for multiple tumor immune activation. Small. 2022;18(45): Article e2203942. 36156383 10.1002/smll.202203942

[143] Bao Y, Ge Y, Wu M, Mao Z, Ye J, Tong W. Record-high ultrasound-sensitive NO nanogenerators for cascade tumor pyroptosis and immunotherapy. Adv Sci. 2023;10(26): Article e2302278. 10.1002/advs.202302278 PMC10502831 37400368

[144] Tang S, Zhou L, He H, Cui L, Ren Z, Tai Y, Xie Z, Cao Y, Meng D, Liu Q, et al. MnO_2_-melittin nanoparticles serve as an effective anti-tumor immunotherapy by enhancing systemic immune response. Biomaterials. 2022;288:121706. 35953328 10.1016/j.biomaterials.2022.121706

[145] Baytar O, Sahin O, Horoz S, Kutluay S. Solar cell efficiency enhancement via mulberry molasses-based carbon quantum dot-supported CdS and CdS-Mn nanomaterials. Waste Biomass Valorization. 2024;15(7):4395–4404.

[146] Rana A, Malik R, Rana M, Kaushik D, Khanna SP, Srivastava R, Suman CK. Studies of optoelectrical properties of Mn-doped ZnO nanostructure for supercapacitor and photodetector applications. J Alloys Compd. 2024;997(7):4395–4404.

[147] Zhang K, Qi C, Cai K. Manganese-based tumor immunotherapy. Adv Mater. 2023;35(19): Article e2205409. 36121368 10.1002/adma.202205409

[148] Yang S-H, Kang B, Choi Y, Rho HW, Son HY, Huh Y-M. Genetic changes and growth promotion of glioblastoma by magnetic nanoparticles and a magnetic field. Nanomedicine. 2021;16(10):787–800. 33890494 10.2217/nnm-2020-0399

[149] Deng Y, Pang J, Ge W, Zhang M, Zhang W, Zhang W, Xiang M, Zhou Q, Bai J. Constructing atomically-dispersed Mn on ZIF-derived nitrogen-doped carbon for boosting oxygen reduction. Front Chem. 2022;10:969905. 36092675 10.3389/fchem.2022.969905 PMC9454009

[150] Horszczaruk E. Properties of cement-based composites modified with magnetite nanoparticles: A review. Materials. 2019;12(2):326. 30669637 10.3390/ma12020326 PMC6356830

[151] Kharat PB, Somvanshi SB, Somwanshi SB, Mopari AM: Synthesis, characterization and hyperthermic evaluation of PEGylated superparamagnetic MnFe_2_O_4_ ferrite nanoparticles for cancer therapeutics applications. In: *Macromolecular Symposia: 2021*. Wiley Online Library; 2021: 2100130.

[152] Somvanshi SB, Thorat ND: Nanoplatforms for cancer imagining. In: *Advances in image-guided cancer nanomedicine.* Bristol (UK): IOP Publishing; 2022, 3-1-3-62.

