# Supplementary material for: Mechanisms and Applications of Manganese-Based Nanomaterials in Tumor Diagnosis and Therapy
Source: Biomater Res. 2025 Feb 28;29:0158. doi: 10.34133/bmr.0158 (PMC11868662; doi:10.34133/bmr.0158)
Supplement: Supplementary 1 — Figs. S1 to S6 [file bmr.0158.f1.zip › Supplementary Fig Caption.docx]

**Supplementary Fig. 1. Mn-based nanomaterials combined with chemotherapy in tumor therapy.**

1. MnO_2_-PDA@Lipo@Geb@Beb; B. Mn-D@BPFe-A; C. MnO_2_-Pt(IV) NPs; D. CD147-MTRN/DOX; Panel A is adapted with permission from Ref. [68], copyright 2022 Drug Delivery. Panel B is adapted with permission from Ref. [69], copyright 2018 Colloids and Surfaces B: Biointerfaces. Panel C is adapted with permission from Ref. [70], copyright 2023 Nanoscale. Panel D is adapted with permission from Ref. [71], copyright 2018 ACS Applied Materials & Interfaces.

**Supplementary Fig. 2. Mn-based nanomaterials combined with radiation therapy in tumor therapy.**

A, Schematic diagram of HGMP NPs for sensitizing radiotherapy; B, Tumor growth curves of 4T1 tumor-bearing mice with different in vivo synergistic treatments over 14 days; NP（[Mn]:2 mg /kg）; RT (6 Gy); PPI (100 mg/kg); C, T1-weighted MR images illustrate the T1 signal intensity of 4T1 tumor-bearing mice before and after intravenous injection with HGMP NPs; D, Data of survival fraction of 4T1 cells colony formation experiments after treatments and exposed with X-ray at different doses. Panel is adapted with permission from Ref. [76], copyright 2023 Acta Biomaterialia.

**Supplementary Fig. 3. Mn-based nanomaterials combined with sonodynamic therapy in tumor therapy.**

1. IR780/PLGA@MnO_2_NPs; B. M-BOC@SP; C. RCMP; Panel A is adapted with permission from Ref. [96], copyright 2022 Frontiers in Bioengineering and Biotechnology. Panel B is adapted with permission from Ref. [97], copyright 2023 Journal of Colloid and Interface Science. Panel C is adapted with permission from Ref. [98], copyright 2023 Advanced Science.

**Supplementary Fig. 4. Mn-based nanomaterials combined with chemodynamic therapy in tumor therapy.**

1. L/D-MnO2@Pt NPs; B. Mn-N/C; C. PPIR780-ZMS; Panel A is adapted with permission from Ref. [101], copyright 2022 Journal of Colloid and Interface Science. Panel B is adapted with permission from Ref. [102], copyright 2024 Advanced Science. Panel C is adapted with permission from Ref. [103], copyright 2022 ACS Nano.

**Supplementary Fig. 5. Mn-based nanomaterials combined with immunotherapy in tumor therapy.**

1. SN/Mn@gHE; B. MnO_2_@OxA@OMV; C. MF@SOR; Panel A is adapted with permission from Ref. [110], copyright 2023 Biomaterials. Panel B is adapted with permission from Ref. [111], copyright 2024 Acta Biomaterialia. Panel C is adapted with permission from Ref. [113], copyright 2024 Journal of nanobiotechnology.

**Supplementary Fig. 6. Mn-based nanomaterials combined with gene therapy in tumor therapy.**

A. Schematic diagram of preparing PEG-MZF-NPs/DDP/CD44-shRNA and magnetic fluid hyperthermia therapy; B. CD44 mRNA expression of ovarian cancer HO8910 cells transfected with CD44-shRNA plasmids; C. Western blotting was conducted to test CD44 protein level; D. Representative images of nude mice bearing xenograft ovarian cancer with various treatments; E. Protein expression of VEGF, survivin, Bcl-2, Bcl-xl, cleaved caspase-3, and cleaved caspase-9 from nude mice xenograft in each treatment group. Panels are adapted with permission from Ref. [115], copyright 2022 Frontiers in oncology.
