# Supplementary figures and images for: Mechanisms and Applications of Manganese-Based Nanomaterials in Tumor Diagnosis and Therapy
Source: Biomater Res. 2025 Feb 28;29:0158. doi: 10.34133/bmr.0158 (PMC11868662; doi:10.34133/bmr.0158)

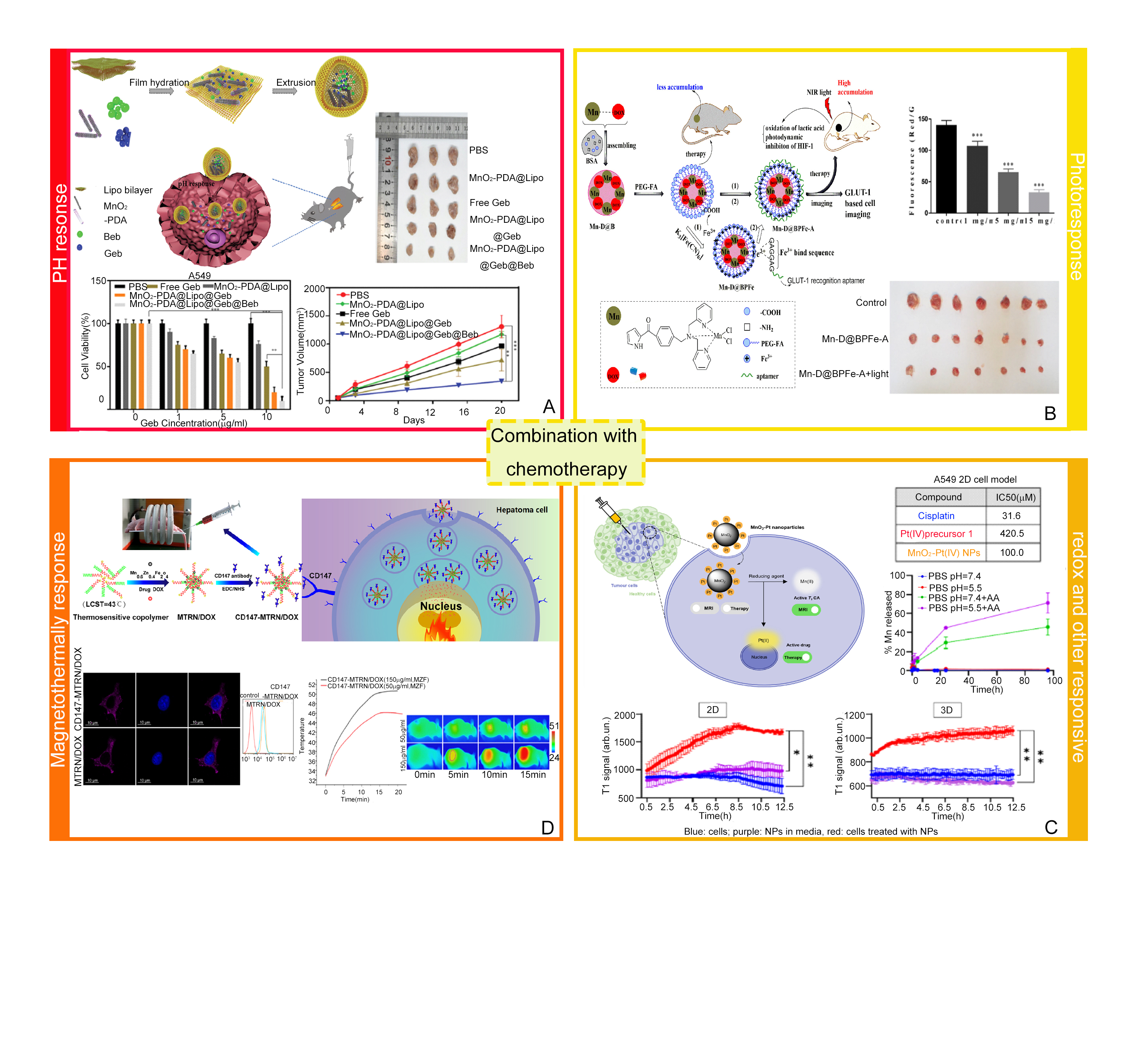

Supplement: Supplementary 1 — Figs. S1 to S6 [file bmr.0158.f1.zip › S1.tif]

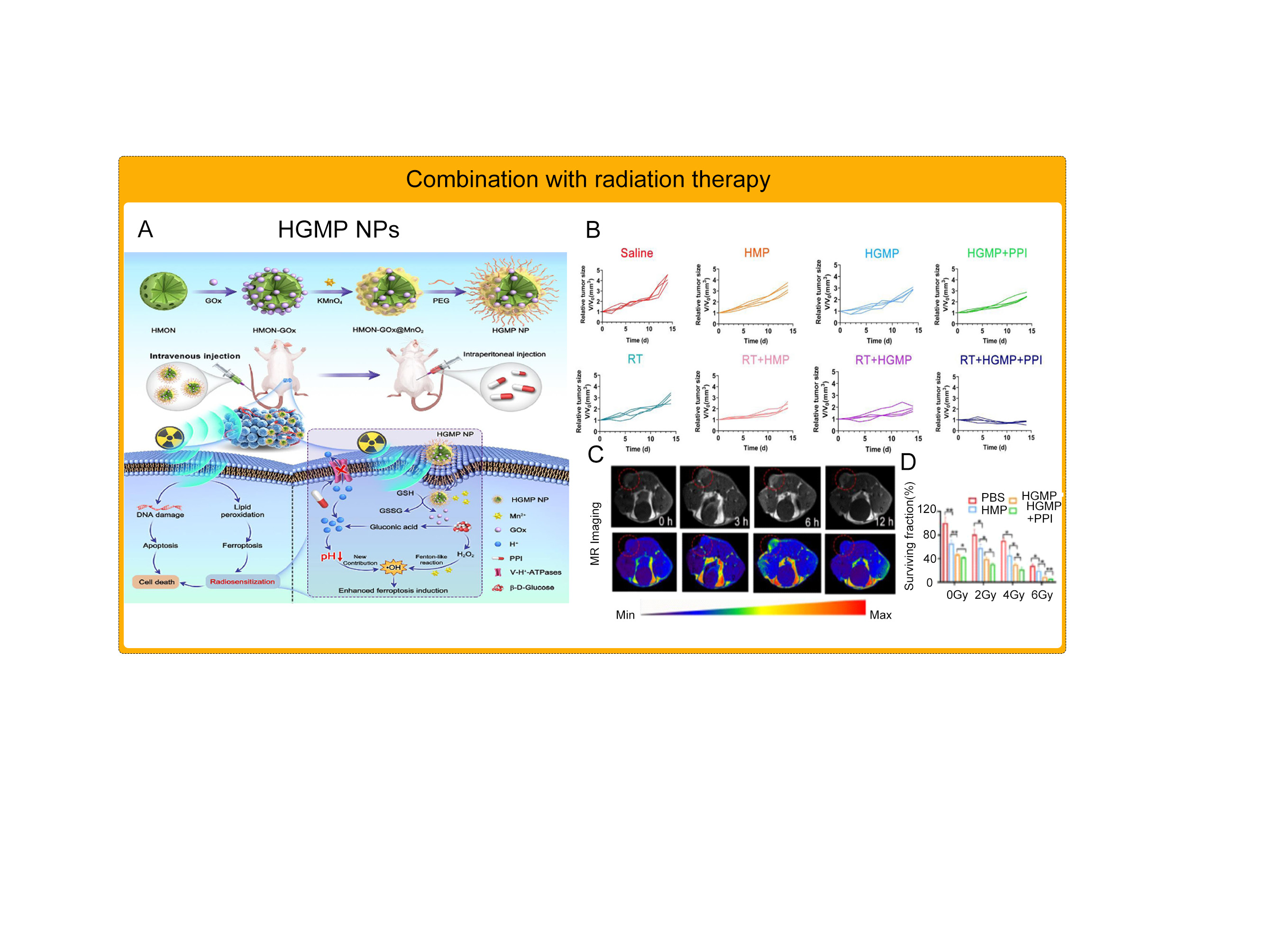

Supplement: Supplementary 1 — Figs. S1 to S6 [file bmr.0158.f1.zip › S2.jpg]

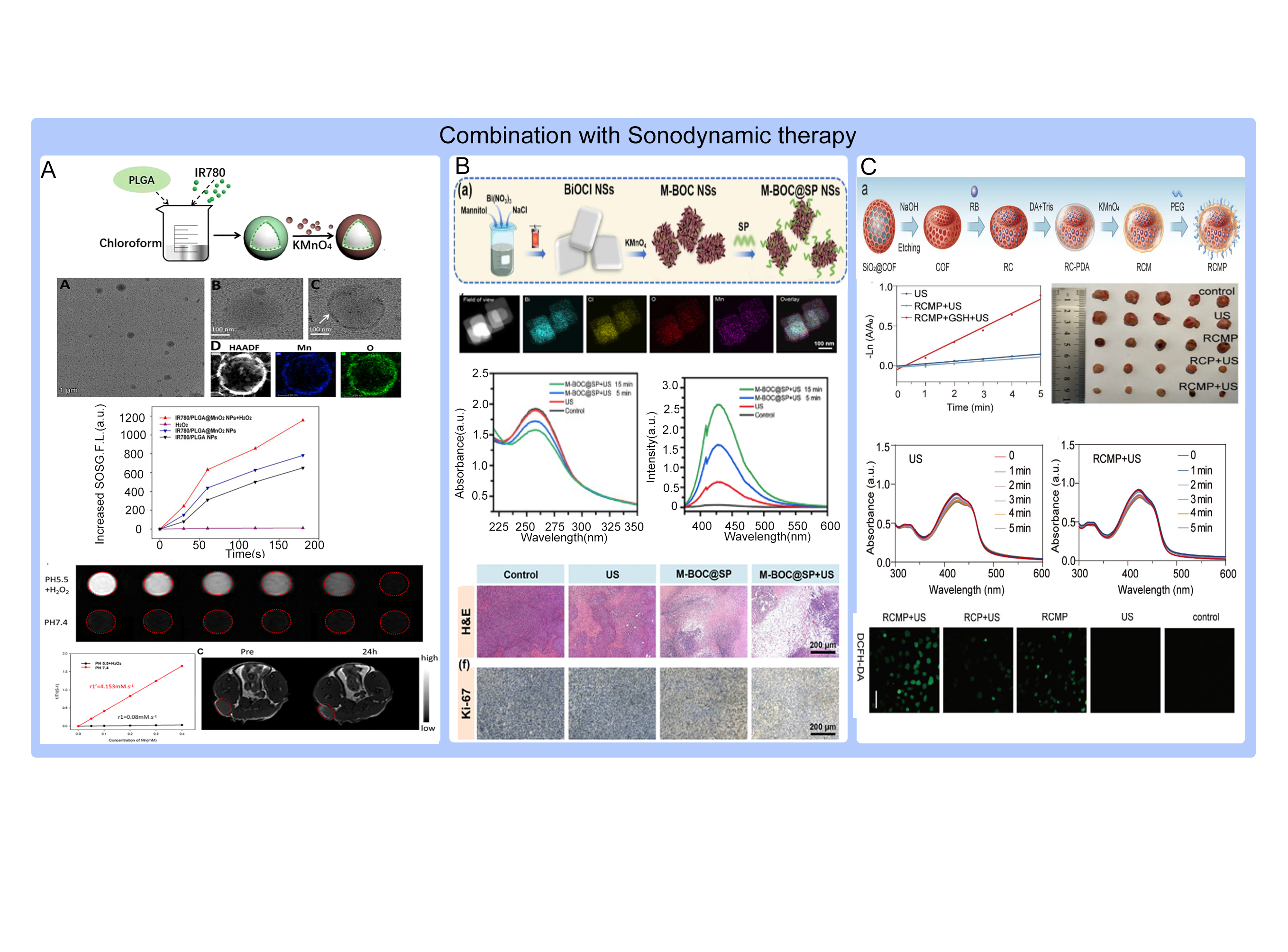

Supplement: Supplementary 1 — Figs. S1 to S6 [file bmr.0158.f1.zip › S3-1.tif]

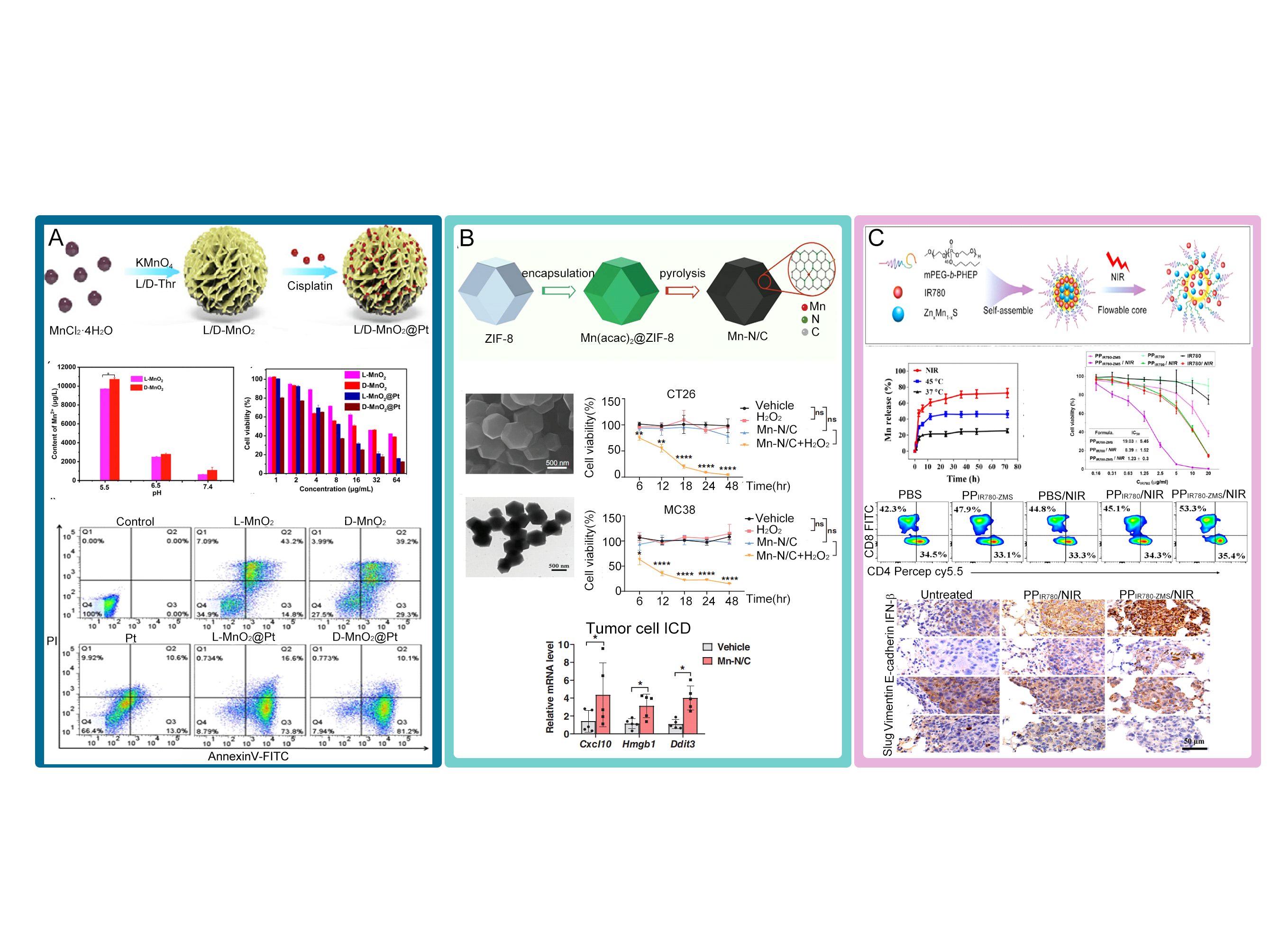

Supplement: Supplementary 1 — Figs. S1 to S6 [file bmr.0158.f1.zip › S4-1.tif]

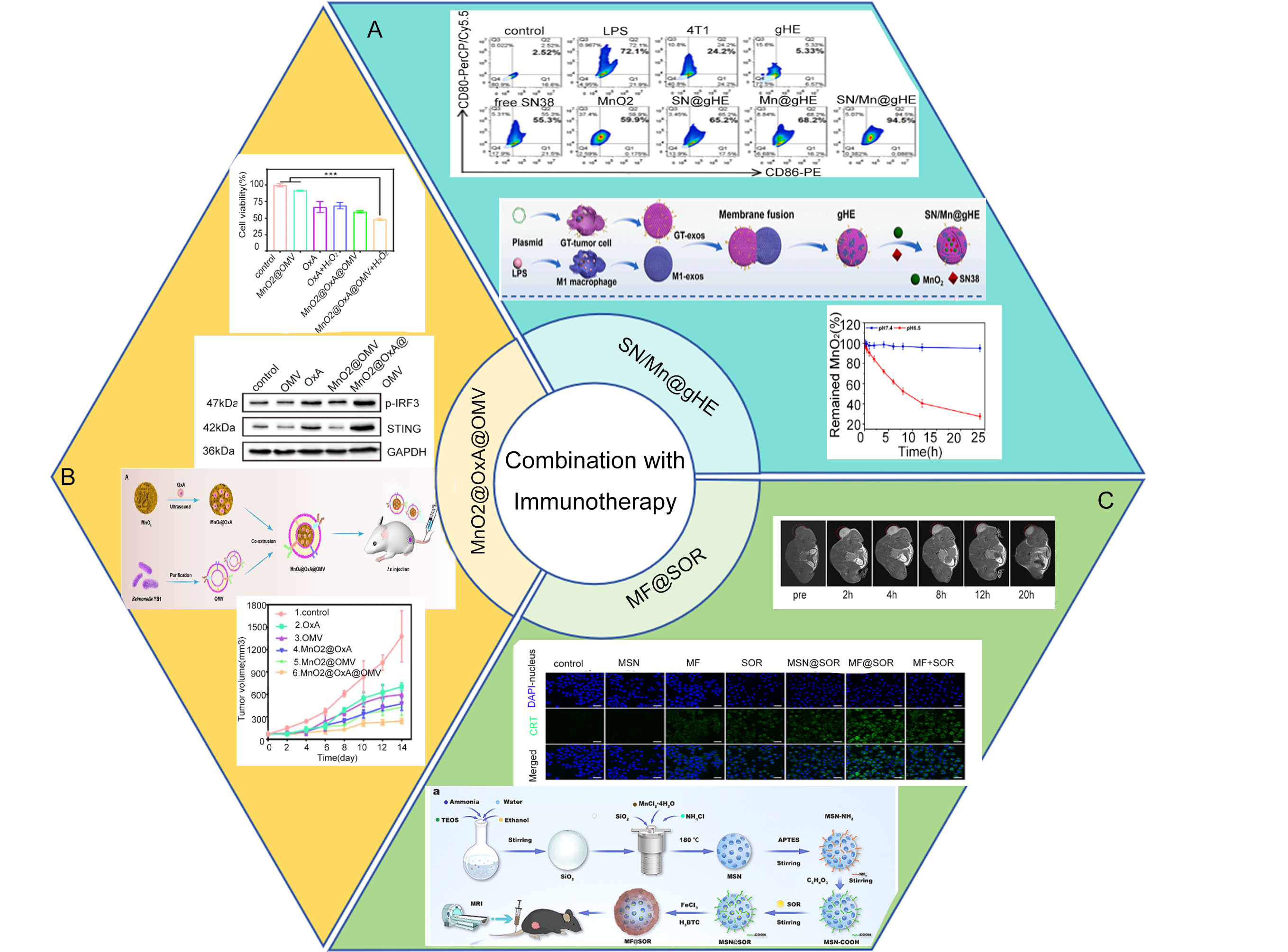

Supplement: Supplementary 1 — Figs. S1 to S6 [file bmr.0158.f1.zip › S5-1.jpg]

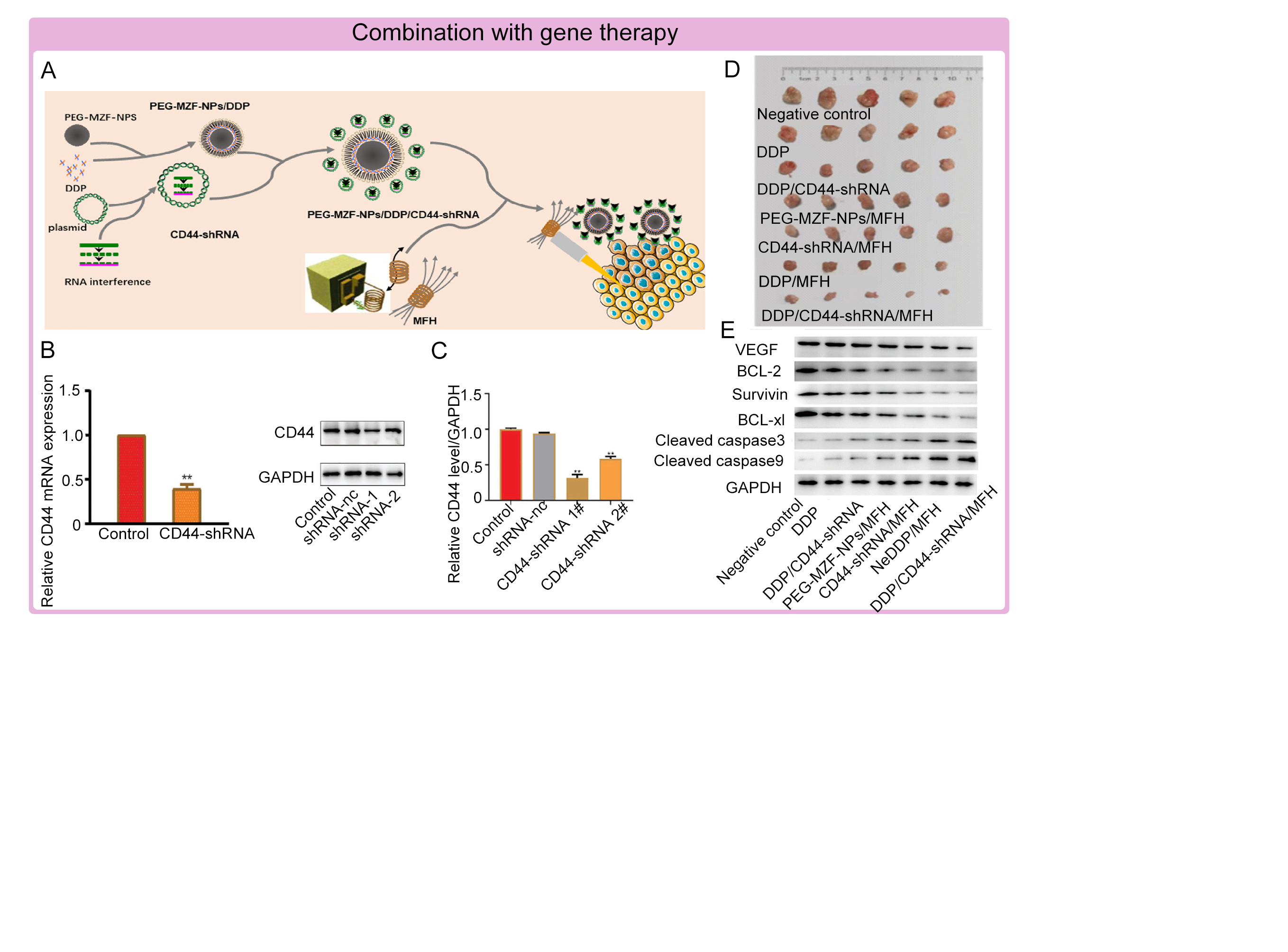

Supplement: Supplementary 1 — Figs. S1 to S6 [file bmr.0158.f1.zip › S6-1.jpg]
